# ERO1A levels are a prognostic indicator in EGFR mutated non small cell lung cancer

**DOI:** 10.1038/s41698-024-00736-1

**Published:** 2024-11-04

**Authors:** M. A. Voronkova, B. Johnson, N. Gandhi, J. M. Koomen, M Patrick, S. Shanthi Bhupathi, V. M. Wu, A. Elliott, A. Vanderwalde, B. Halmos, L. A. Hazlehurst

**Affiliations:** 1 West Virginia University Cancer Institute, Morgantown, WV USA; 2 Caris Life Sciences, Phoenix, AZ USA; 3 Molecular Oncology, Moffitt Cancer Center, Tampa, FL USA; 4 Penn State Cancer Institute, Penn State College of Medicine, Hershey, PA USA; 5 Montefiore Einstein Comprehensive Cancer Center, Bronx, NY USA; 6 Department of Pharmaceutical Sciences, West Virginia University, Morgantown, WV USA; 7 Department of Medical Oncology, West Virginia University School of Medicine, Morgantown, WV USA

**Keywords:** Diagnostic markers, Cancer microenvironment

## Abstract

We have identified endoplasmic reticulum oxidoreductase 1 alpha (*ERO1A*) as a poor prognostic indicator in epidermal growth factor receptor (EGFR)-mutated non-small cell lung cancer (*EGFR*^MUT^-NSCLC). In addition, comparison of high versus low *ERO1A* expression among cohorts of *EGFR*^MUT^-NSCLC primary samples revealed that ERO1A expression correlated with increased expression of proteins that regulate secretion. Using the CPTAC proteomic data set in lung adenocarcinoma we found that high ERO1A protein expression correlated with both extracellular matrix and matrix modifying enzymes. In this report, we found that ablating *ERO1A* expression was a determinant of clonogenicity, tumor sphere formation, spheroid growth and growth in vivo, as well as response to Osimertinib. We validated that *ERO1A*-knockout *EGFR*^MUT^-LUAD cell lines demonstrated a reduction in secretion of both laminin gamma 2 (LAMC2) and the collagen modifying enzyme lysyl oxidase-like 2 (LOXL2). Our work supports the role of *ERO1A* in modulating the tumor microenvironment that is likely to contribute to tumor progression.

## Introduction

*EGFR*^MUT^-NSCLC typically responds well to EGFR inhibitors but unfortunately the emergence of drug resistant disease remains an obstacle; thus, novel strategies are required to improve patient outcomes. *ERO1A* has recently emerged as an interesting putative target for the treatment of cancer. *ERO1A* expression has been shown to be a poor prognostic indicator across multiple tumor types including lung, pancreatic, hepatocellular carcinoma and multiple myeloma^1–6^. ERO1A, in conjunction with protein disulfide isomerase (PDI), is critical for the formation of disulfide bonds in target proteins that are processed in the endoplasmic reticulum (ER). ERO1A accepts electrons from reduced PDI resulting in oxidative folding of PDI^7,8^. ERO1A is capable of forming de novo disulfide bonds utilizing Flavin adenine dinucleotide (FAD) as cofactor leading to the generation of hydrogen peroxide (H_2_O_2_). In mammals, *ERO1A* expression is not essential for oxidative protein folding due to the expression of other oxidases including *ERO1B*, peroxiredoxin 4 (*PRDX4*) and glutathione peroxidase (*GPX7*/*8*)^9,10^. Despite the redundancy of enzymes capable of contributing to oxidative folding within the ER, experimental evidence continues to support the critical role of *ERO1A* expression in the progression of cancer^11,12^.

Recent studies have indicated that *ERO1A* influences multiple pathways including (i) lipid metabolism^13^, (ii) response to inhibition of eIF2α,a regulator of ER stress^14^ (iii) upregulation of the β-catenin pathway^15^, (iv) promotion of IL-6 signaling^16^ (v) secretion of VEGF^17^ and (vi) and growth using in vitro and in vivo models^4^. Due to the function of protein folding in the ER, it is not surprising that genetic knockdown of *ERO1A* in cancer cells results in a myriad of effects on signaling. Recently, Varone et al. demonstrated that depletion of ERO1A in HeLa cells resulted in increased N-glycosylation of VEGF, which inhibited its secretion^17^.

Secreted Laminin 332 is comprised of LAMC2, laminin alpha 3 (LAMA3) and laminin beta 3 (LAMB3). Laminin 332 is a high affinity receptor for integrins A6B1, A6B4 and A3B1, and genetic manipulation of levels of *LAMC2* is known to control cell migration, as well as contribute to the stem cell niche and an immune-suppressive tumor microenvironment^18–20^. LOXL2 is an extracellular copper-dependent amine oxidase that crosslinks collagens and elastin to modify the stiffness of the matrix. Recently LOXL2 was shown to contribute to cell surface matrix remodeling, and contributing to collective dissemination of free-floating aggregates^21^. In addition, LOXL2 was shown to be important in adhesion-mediated drug resistance^22^. In this manuscript, we explore the relationship between *ERO1A* expression and the critical determinants of progression of *EGFR*^MUT^-LUAD, including colony and tumor sphere formation, spheroid growth, matrix secretion and sensitivity to EGFR inhibitors.

## Results

### *ERO1A* expression is a poor prognostic indicator of response in NSCLC

**Fig. 1:**
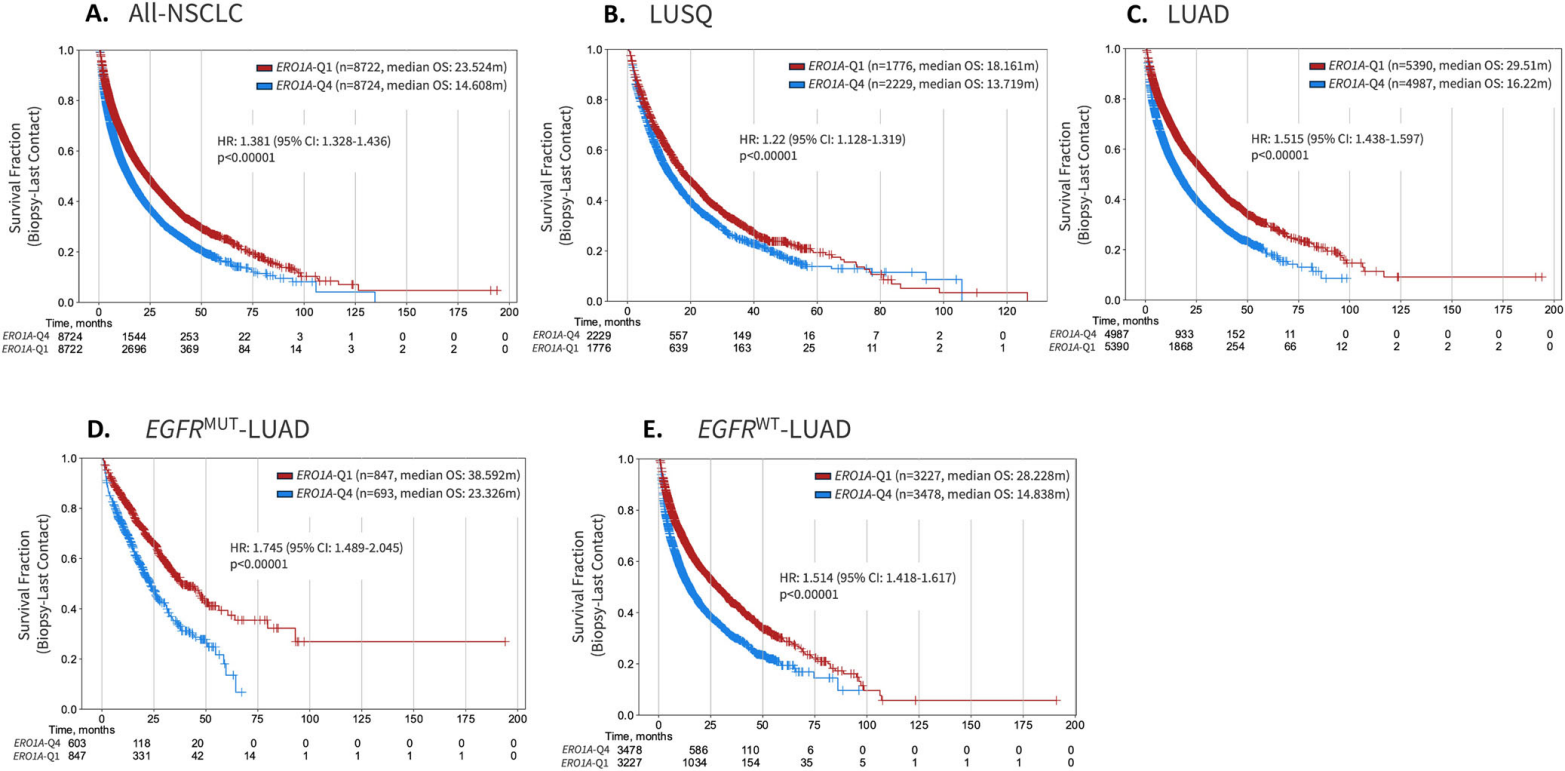
*ERO1A* is associated with poor prognosis in NSCLC. High expression of *ERO1A* is associated with poor prognosis in all-NSCLC(**A**), when considering squamous (**B**) and adenocarcinoma histology (**C**) and when accounting for *EGFR* mutation status (**D**, **E**). ERO1A quartiles are based on expression in all-NSCLC patients and were consistently applied across all subtypes of NSCLC.

**Fig. 2:**
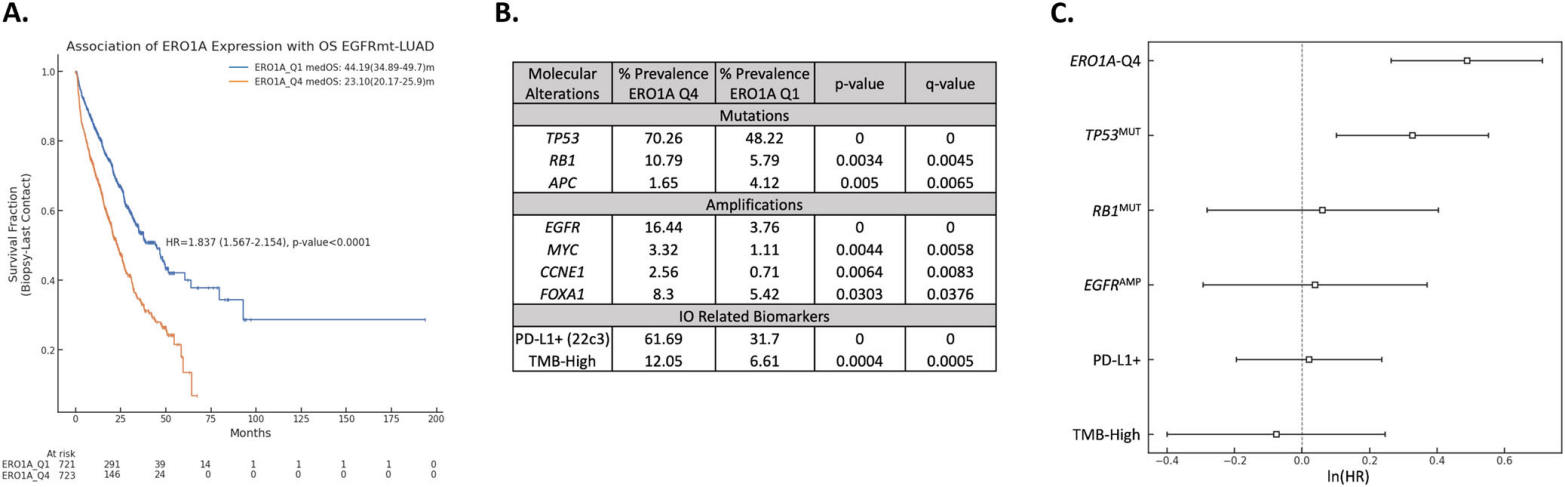
Multivariate analysis to test the association of ERO1A expression with overall survival (OS). ERO1A quartile cutoffs were determined based on the expression of ERO1A in EGFRmt-LUAD. **A** Patients with tumors expressing high ERO1A levels have increased survival. **B** Molecular alterations associated with ERO1A expression prevalent in either cohort at ≥2% and statistically significantly different are shown in the table. **C** Molecular alterations with a prevalence difference of at least 5% between the cohorts were incorporated as co-variates in a complete-case multivariate analysis. High ERO1A expression and TP53 mutations were independently associated with poor OS in EGFRmt-LUAD (*n* = 960).

**Fig. 3:**
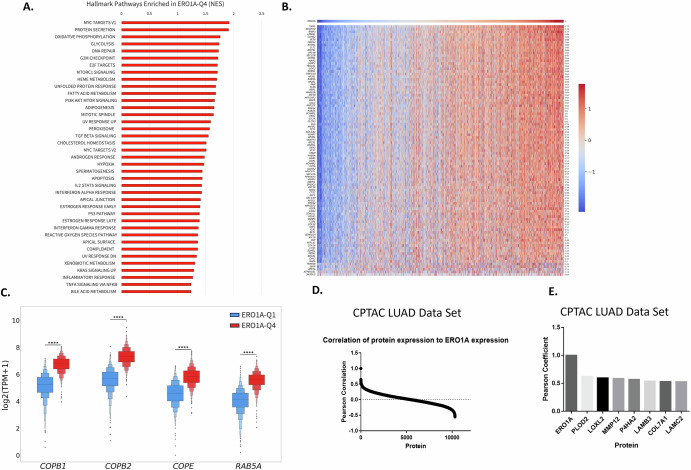
*ERO1A* levels correlate with changes in protein secretion. High expression of *ERO1A* was associated with an enrichment of a number of Hallmark Pathways as revealed by GSEA. The second most significant pathway was associated with regulating protein secretion (**A**). The association of *ERO1A* expression was established with the expression of genes involved in Hallmark protein secretion pathway. Genes were sorted based on their Spearman correlation with *ERO1A* and are listed to the right side of the heatmap (**B**). Subset of genes involved in vesicle trafficking from ER to Golgi as well as regulation of exosomes were investigated and observed to be significantly enriched in *ERO1A*-Q4 tumors (**C**). Correlation between ERO1A and levels of other proteins downloaded from CPTAC, a publicly available lung adenocarcinoma tumor proteomics dataset, were analyzed using LinkedOmics (http://linkedomics.org/data_download/CPTAC-LUAD/)^28^ (**D**). 7 out of 28 targets with highest correlation to ERO1A levels are extracellular matrix proteins (**E**).

Using the CARIS dataset of NSCLC patient specimens, we asked whether *ERO1A* expression was associated with overall prognosis in NSCLC. High *ERO1A* expression was associated with poor survival with a continuum of response being noted across all quartiles (Supplemental Fig. 1). To demonstrate the strongest phenotypic association, data was analyzed comparing the top (Q4) and bottom (Q1) quartile of *ERO1A* expression. As shown in Fig. 1A, *ERO1A*-Q4 was associated with poor prognosis across all NSCLC samples, with a median OS = 14.61 m (vs *ERO1A*-Q1 median OS = 23.524 m, HR = 1.381, *p* < 0.00001). *ERO1A*-Q4 was also associated with poor prognosis in both squamous (HR = 1.22) and adenocarcinoma (HR = 1.51) histology, with a median difference in survival of 4.4 m and 13.3 m, respectively (Fig. 1B, C). To determine whether specific driver mutations influenced the prognosis associated with *ERO1A* expression, we examined *EGFR* mutant- and wild-type- LUAD, in which *ERO1A*-Q4 was observed with poor prognosis in both genotypes, with a HR of 1.745 and 1.515, respectively (both *p* < 0.00001). Similar results were observed in *KRAS* mutant- LUAD (Supplemental Fig. 2). Together these data indicate that high *ERO1A* expression is associated with poor prognosis in LUAD independent of histology or known driver alteration. Examining data via the LinkedOmics platform, ERO1A protein expression was high in LUAD (Supplemental Fig. 3) compared to other indications and was found to be higher in tumor versus normal lung tissue. To focus in on a specific subtype that is uniformly treated with kinase inhibitors, we investigated in more depth the role of *ERO1A* in the progression of *EGFR*^MUT^-LUAD. A multi-variable analysis was performed to determine whether ERO1A was an independent marker of poor prognosis in EGFR mutant NSCLC. As shown in Fig. 2A–C, ERO1A high levels and TP53 mutations were independently associated with poor overall survival. Interestingly, 70 percent of patients expressing high levels of ERO1A also harbored p53 mutations. Despite this correlation ERO1A levels and TP53 mutations remained an independent prognostic indicator in EGFR driven NSCLC. Gene set enrichment analysis revealed an enrichment of pathways involved in proliferation, unfolded protein response, metabolism and immune/inflammation in *ERO1A*-Q4 tumors (Fig. 3A). Similar to what others have reported, *ERO1A* expression correlated with changes in Hypoxia and the Unfolded Protein Response. However, in addition to these pathways, protein secretion was one of the most enriched pathways in *ERO1A*-Q4 tumors. As shown in Fig. 3B, increased expression of genes involved in the protein secretion pathway appear tightly correlated with increased expression of *ERO1A*. Moreover, several proteins involved in vesicle transport from the ER to Golgi, including but not limited to *COPB2*, *COPB1*, *COPE* (all part of the coatomer complex) and *RAB5A* (contributes to endocytic vesicle formation, as well as exosome secretion^23^), showed significantly increased expression in the *ERO1A*-high samples (Fig. 3C). Using the publicly available primary LUAD CPTAC data (http://linkedomics.org/data_download/CPTAC-LUAD/), we correlated expression of 10,315 detected proteins to ERO1A expression across 110 LUAD patient samples (Fig. 3D). These data are based on LC-MS/MS quantification in tumor tissues, and thus, we anticipate that less abundant proteins, such as chemokines and cytokines, may be under-represented in this data set. Several of the top hits would be predicted to change the local matrix associated with the tumor microenvironment, as 7 out of the top 28 most strongly correlated proteins were matrix-related proteins (Fig. 3E).

### Depletion of ERO1A inhibits clonogenicity and tumor sphere formation in HCC4006 and PC-9 cells

**Fig. 4:**
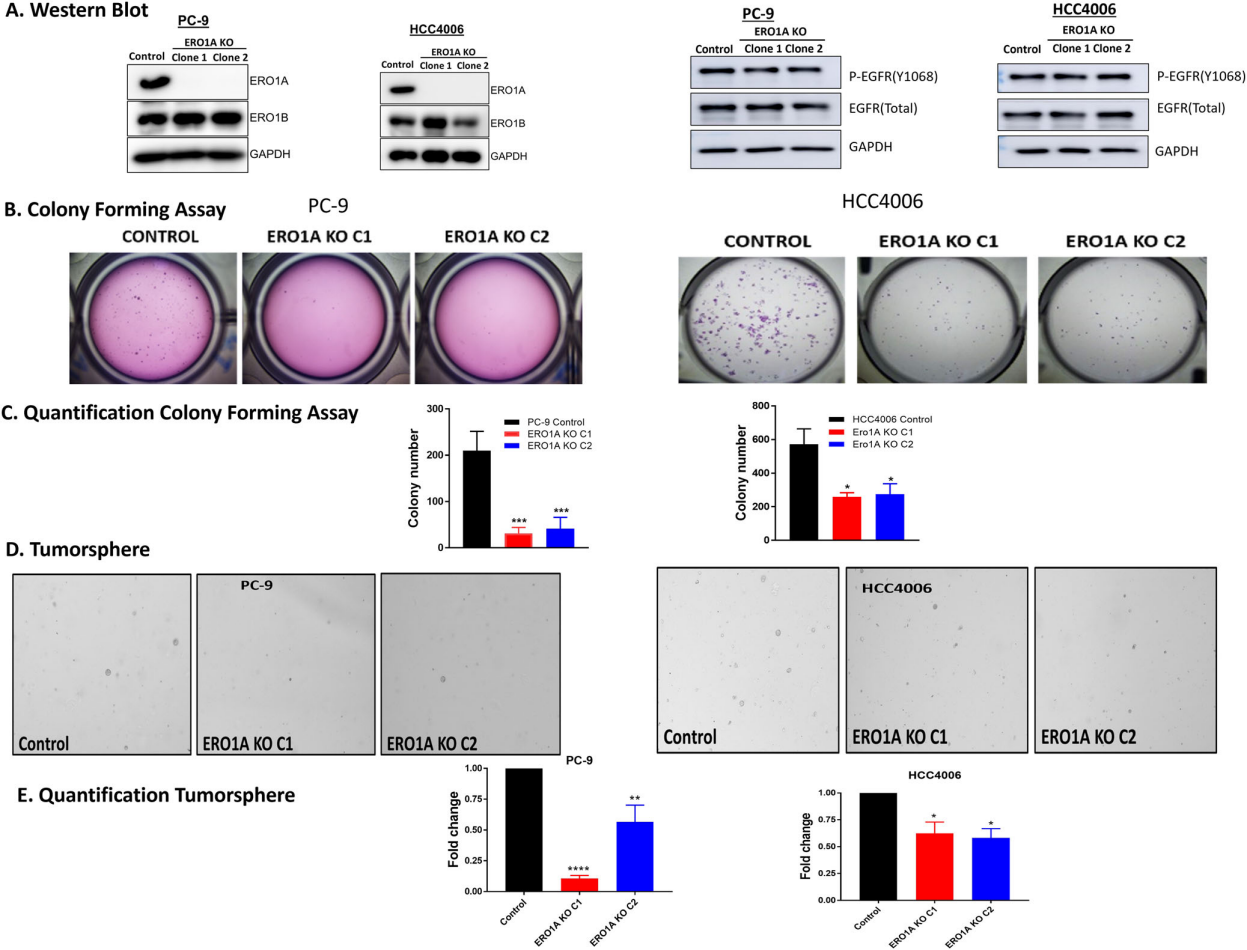
Depletion of ERO1A does not alter EGFR activation, yet decreases clonogenicity and tumor sphere formation. Shown is a representative image demonstrating CRISPR deletion of *ERO1A* in PC-9 and HCC006 cell line. ERO1B expression levels do not compensate for loss of ERO1A. Representative western blots demonstrate that *ERO1A* KO does not affect total levels of EGFR or p-EGFR in two *EGFR*^MUT^-NSCLC cell lines (**A**). *ERO1A* KO cells form smaller number of colonies in soft agar and on tissue culture plates. Representative fields of view from one independent experiment are shown. ***p = 0.0004 by one-way ANOVA, *p = 0.0111 by repeated measures ANOVA, *n* = 3–4 independent experiments performed in triplicates (**B**, **C**). *ERO1A* KO clones reveal a decrease in total tumor sphere formation in both PC-9 and HCC4006 cell lines. Representative fields of view from one independent experiment are shown. Shown is the means of 3-4 independent experiments performed in triplicates. Numbers are mean ± SEM. ****p < 0.0001, **p = 0.0072, *p < 0.05; Dunnett’s multiple comparisons test following one-way ANOVA (**D**–**E**).

To determine whether *ERO1A* expression was causative for phenotypes predicted to contribute to tumor progression, as well as secretion of matrix, two *ERO1A* knockout *EGFR*-mutated isogenic cell line models, referred to as HCC4006 and HCC4006 *ERO1A* KO and PC-9 and PC-9 *ERO1A* KO, were established (Fig. 4A). In addition, to ensure rigor, two independent clones of the *ERO1A* KO cells were established to determine whether the phenotype is observed across multiple cell lines and clones. We initially asked whether depletion of ERO1A using CRISPR strategies altered the levels of ERO1B, which enzymatically is considered functionally redundant with respect to the capacity to oxidize PDI. As shown in Fig. 4A, depletion of ERO1A had no effect on ERO1B levels in either HCC4006 or PC-9 *EGFR*-driven cell lines. Moreover, lack of ERO1A expression did not alter the phosphorylation status or levels of total EGFR protein (Fig. 4A). In addition, depletion of ERO1A did not alter activation of MAPK or AKT (Supplemental Fig. 4A, B) In some contexts, depletion of ERO1A levels leads to ER stress in cancer cell lines in vitro and in vivo^3,14^. However, depletion of ERO1A was not sufficient to induce markers of ER stress in these *EGFR* driven cell lines (Supplemental Fig. 4C, D) in cell culture. We further assessed growth in tissue culture and interestingly HCC4006 cells depleted of ERO1A showed a significant decrease (*p* < 0.05, two-way repeated ANOVA) in cell growth while PC-9 showed a tend in decreased growth but was not statistically significanct (*p* > 0.05, two-way repeated ANOVA) (Supplemental Fig. 5A, B). As shown in Supplemental Fig. 5C, D, depletion of ERO1A was sufficient to increase sensitivity to Osimertinib inhibition in both cell lines. For HCC4006 cells, the *ERO1A* KO lines demonstrated a significant 98 and 131 100-fold decrease in IC50 values in clone 1 and clone 2, respectively compared to control cells (*p* < 0.05, Students *t*-test, *n* = 4 independent experiments). In contrast in PC-9 cells ERO1 KO clone 1 demosntrated a 2.58 fold lower IC50 value (*p* > 0.05, Students *t*-test, *n* = 4 independent experiments) while ERO1A KO clone 2 demonstrated a 22 fold dcrease in IC50 value (*p* < 0.05, Students *t*-test *n* = 4 idependent experimenst). Examining clinical samples, EGFR mutated NSCLC patients with tumors with high ERO1A expression experienced a shorter time on Osimertinib treatment (*p* < 0.05, log rank). However, ERO1A was not an independent marker predictive marker of time on treatment (Supplemental Fig. 6A–C). Further studies are required to determine the difference in change of sensitivity to Osimertinib between PC-9 and HCC4006 ERO1A depleted cell line. Indeed these cell line models may provide for isogenic systems to identify additional markers of dependency of ERO1A in predicting response to Osimertinib treatment in clinical samples. Despite no change in growth on plastic, PC-9 cells showed a significant decrease in colony formation in soft agar (*p* < 0.05, ANOVA, Fig. 4C). For HCC4006 cells, clonogenicity was determined at low cell density and counting adherent colonies on plastic, where depletion of ERO1A resulted in a significant decrease in the number of colonies compared to control cells (Fig. 4C). To address whether depletion of ERO1A expression is critical for tumor sphere formation as a hallmark of fitness of the cancer stem-like cell population, a tumor sphere assay was performed using low attachment plates. Depletion of ERO1A in both cell lines significantly (*p* < 0.05, ANOVA) inhibited tumor sphere formation (Fig. 4D). Together, these data suggest that depletion of ERO1A inhibits properties of the stem-like phenotype (colony and spheroid formation) independent of EGFR activation status, which was consistent in two independent *EGFR*-driven cell lines.

### ERO1A regulates secretion of LAMC2 and LOXL2

Based on the function of ERO1A in mediating oxidative folding of proteins within the ER, we asked whether ERO1A protein expression correlates with proteins of interest that could modulate the cancer stem-like phenotype. Fuji et al. previously reported that ERO1A was critical for secretion of collagen^24^ from hepatic stellate cells and double-mutant ERO1A and ERO1B knockout mice show decreased connective tissue in the tail, a finding that correlated with decreased collagen deposition^25^. Our data indicate that *ERO1A*-high samples have increased expression of the machinery for protein secretion and increased expression of proteins associated with matrix remodeling. Together, these data indicate that ERO1A may be an important regulator of secretion of matrix in cancer cells. Of interest to our laboratory was the finding that ERO1A protein expression correlated with LAMC2 and LAMB3 (Fig. 3E) protein expression, both of which are components of Laminin 332, a matrix protein that has been shown to support the cancer stem cell phenotype and, similar to ERO1A, is reported to be a poor prognostic indicator of survival in cancer^26,18^. In addition, LOXL2, a member of the lysyl oxidase family, which is a secreted copper-dependent amine oxidase and catalyzes the covalent crosslink of collagen and elastin was of interest to further investigate.

**Fig. 5:**
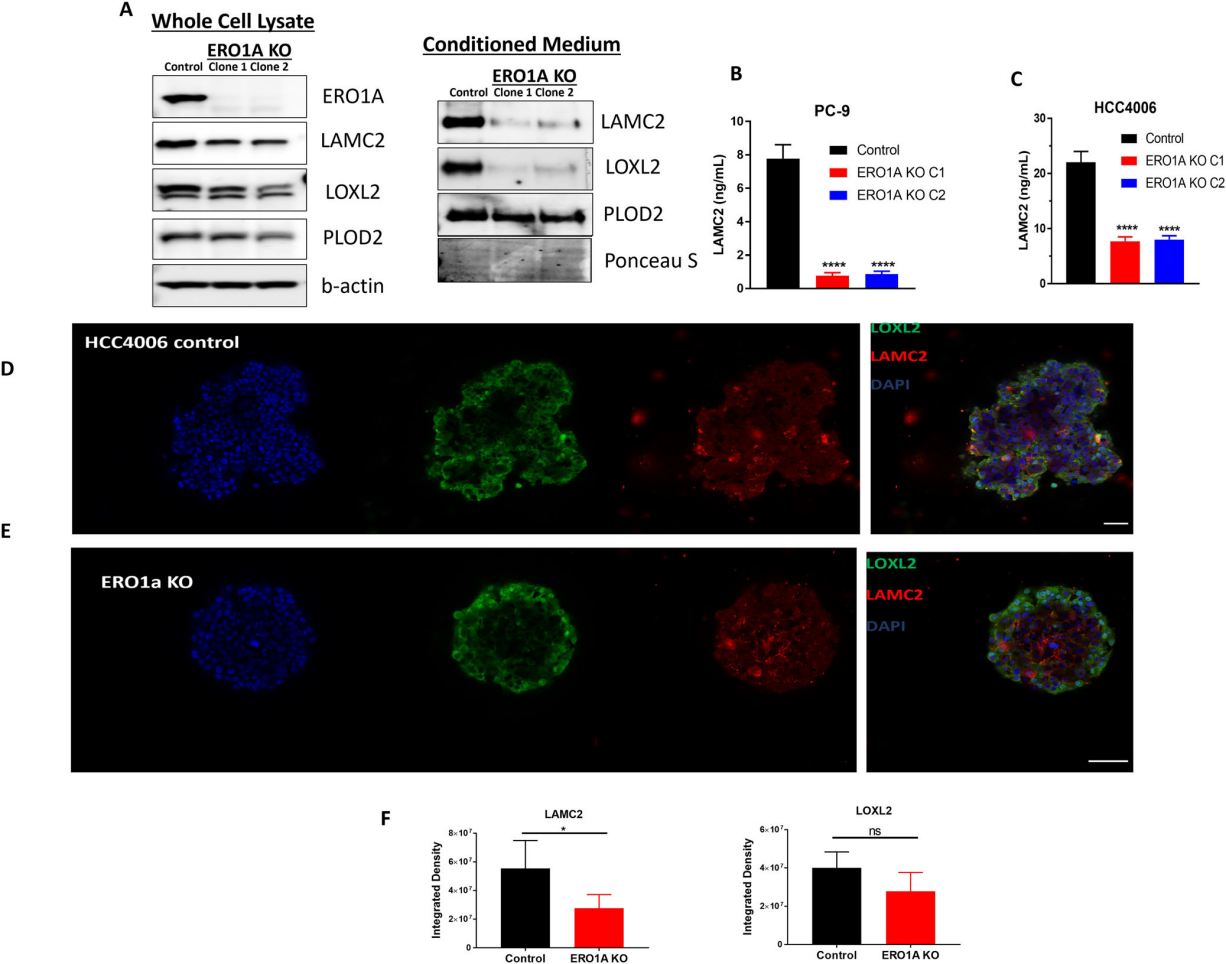
ERO1A protein levels correlate with levels of LOXL2 and LAMC2 protein levels. Levels of LAMC2 and LOXL2 are dramatically reduced in conditioned medium collected from *ERO1A* KO cells. Representative western blots on whole cell lysates and conditioned media in PC-9 cell line are shown (**A**). Concentration of LAMC2 secreted into conditioned media by *ERO1A* KO cell lines is reduced compared to control cells. ELISA was performed in triplicate and the average ± S.D. of *n* = 3 combined results are shown. ****p < 0.0001; Dunnett’s multiple comparisons test following one-way ANOVA (**B**–**C**). Representative images of immunofluorescence staining for LOXL2 and LAMC2 in HCC4006 spheroids, scale bar - 50 µm (**D**–**E**). Quantification of LOXL2 and LAMC2 immunofluorescent staining in control and *ERO1A* KO HCC4006 cells cultured as tumor spheres. *p = 0.0209 by two-tailed unpaired *t*-test, *n* = 5, experiment was repeated 3 times. ns – not significant (**F**).

To determine whether ERO1A expression is causally related to expression or secretion of LAMC2 or LOXL2, we collected conditioned media (CM) from control and cells in which *ERO1A* was knocked out. As shown in Fig. 5A, both LOXL2 and LAMC2 were reduced in CM derived from PC-9 cells. In addition, ELISA based quantification of LAMC2 levels in CM revealed a significant decrease (*p* < 0.05, ANOVA) in both HCC4006 and PC-9 cells depleted of ERO1A compared to control cells (Fig. 5B, C). Finally, we asked whether LAMC2 and/or LOXL2 expression is reduced in HCC4006 cells, which form spheroids when cultured in low attachment plates. We observed that depletion of ERO1A in HCC4006 cells resulted in small and more well-defined margins compared to the control HCC4006 cell line. In addition, a significant reduction in LAMC2 was found and a trend towards a decrease in LOXL2 was observed in the *ERO1A* KO HCC4006 cells compared to the HCC4006 control cells (Fig. 5E, F).

### Effects of conditioned media on *ERO1A* knockout phenotypes

**Fig. 6:**
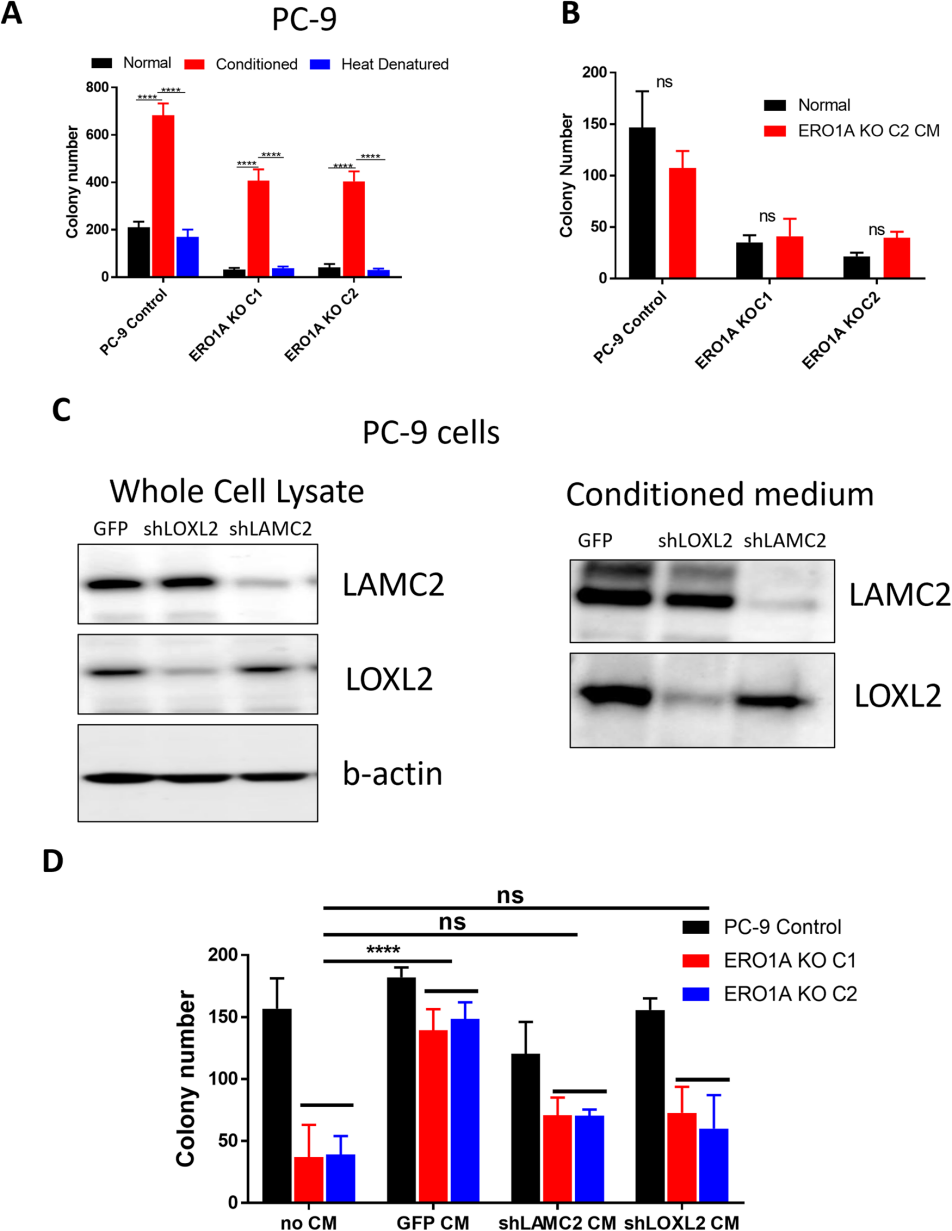
Tumorigenic properties of ERO1A are mediated in part by a secreted factor. Soft agar colony formation assay using regular non-conditioned medium (Normal), medium conditioned by control cells (Conditioned) and conditioned medium from control cells after heat denaturation (Heat denatured), ****p < 0.0001, two-way ANOVA followed by Sidak’s multiple comparisons test, *n* = 3 (**A**). Conditioned medium generated by *ERO1A* KO cells does not rescue colony formation abilities of *ERO1A* KO cells (**B**). Representative immunoblot showing level of LAMC2 and LOXL2 knockdown in PC-9 cells. LAMC2 or LOXL2 knockdown dramatically decrease levels of secreted LAMC2 and LOXL2, accordingly (**C**). Conditioned medium generated by sh*LOXL2* and sh*LAMC2* PC-9 cells partially rescue colony formation abilities of *ERO1A* KO cells in soft agar assay. GFP—empty vector control. Data are means ± SD. ****p < 0.0001, Tukey’s multiple comparisons test following two-way ANOVA. ns – not significant (**D**).

To determine whether secreted factors were responsible for reduction in clonogenicity in PC-9 cells, CM was collected from control cells and used to perform soft agar analysis. CM derived from control cells was able to partially rescue a reduction in colony number of PC-9 *ERO1A* KO cells (Fig. 6A). CM which was heat denatured was unable to rescue the reduction in clonogenicity observed in *ERO1A* KO cells. In addition, CM derived from *ERO1A* KO cell line was unable to rescue the reduction in clones observed in PC-9 cells depleted of ERO1A (Fig. 6B). Together, these data indicate that a secreted factor, whose activity can be disrupted by heat denaturation, is decreased in cells with ERO1A depletion and contributes to soft-agar clonogenic fitness. We next sought to determine whether depletion of either LAMC2 or LOXL2 using shRNA strategies in the control cell line was sufficient to inhibit the rescue of *ERO1A* KO cells exposed to CM. shRNA strategies effectively reduced the expression of either LAMC2 or LOXL2 in CM collected from PC-9 control cells. Reducing the levels of LAMC2 or LOXL2 reduced the rescue of the *ERO1A* KO cells from 4-fold to ~2- and 3-fold, respectively, albeit these findings were not statistically significant when compared to the controls without CM (Fig. 6D). Together, these data indicate that the role of ERO1A in modulating the secretome is likely multifactorial but has the potential to shift the surrounding tumor microenvironment in favor of growth.

### Depletion of ERO1A reduces tumor growth in vivo

**Fig. 7:**
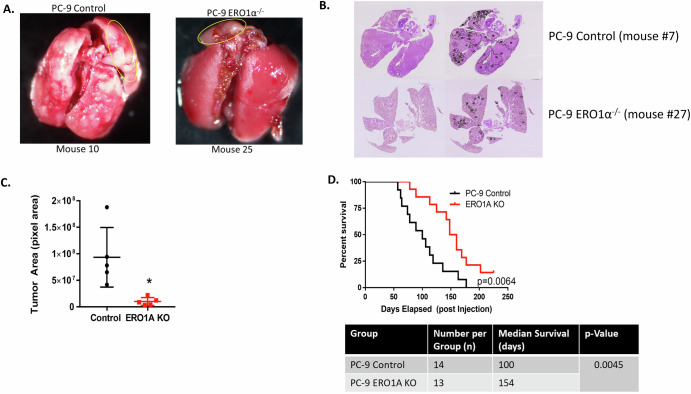
*ERO1A* expression is associated with increased tumor burden and decreased overall survival in SCID-Beige mice bearing *EGFR*^MUT^-LUAD tumors. Picture of tumors formed and H & E staining from the lungs of ERO1A replete and KO mice. H & E was performed on 5 random lungs from each group selected by a random number generator and tumor margins quantified using ImageJ (**A**, **B**). Each data point represents the average on three sections from each mouse. *p = 0.0110, unpaired *t*-test (**C**). Kaplan–Meier curve showing that *ERO1A* KO increased overall survival compared to the control group. *p* = 0.0064, log-rank (Mantel-Cox) test (**D**).

To determine whether ERO1A depletion has an impact in tumor engraftment and progression, PC-9 control and *ERO1A* KO cells (which showed no change in growth in plastic) were injected into SCID-BIEGE mice. Depletion of ERO1A resulted in less overall tumor burden and reduction in number of nodules (Fig. 7A–C). The reduction in tumor burden corresponded to an increase in survival of mice injected with ERO1A depleted cells (*p* < 0.05 Log Rank Test) compared to mice injected with control PC-9 cells. Together, these data genetically validate the potential importance of targeting ERO1A for the treatment of *EGFR*^MUT^-NSCLC and provide rationale to further understand the mechanism underpinning the role of *ERO1A* in meditating the progression of *EGFR*^MUT^-NSCLC.

## Methods

### Next-generation sequencing (NGS) of DNA

A total of 36,657 NSCLC tumors were molecularly profiled at Caris Life Sciences (Phoenix, AZ) between 2017–2022. NGS was performed on genomic DNA isolated from formalin-fixed paraffin-embedded (FFPE) tumor samples using the NextSeq or NovaSeq 6000 platform (Illumina, Inc., San Diego, CA). For the Nextseq sequenced tumors, a custom-designed SureSelect XT assay was used to enrich 592 whole-gene targets (Agilent Technologies, Santa Clara, CA). Further, for the NovaSeq sequenced tumors, 719 clinically relevant genes were enriched at a high coverage and read-depth, along with another panel designed to enrich for >20,000 genes at lower read-depth. All variants were detected with >99% confidence based on allele frequency and amplicon coverage, with an average sequencing depth of coverage of 800 and an analytic sensitivity to identify variants with a variant allele frequency of ≥5%. Genetic variants identified were interpreted by board-certified molecular geneticists and categorized as ‘pathogenic (P),’ ‘likely pathogenic (LP),’ ‘variant of unknown significance,’ ‘likely benign,’ or ‘benign,’ according to the American College of Medical Genetics and Genomics (ACMG) standards. When assessing mutation frequencies of individual genes, ’pathogenic’ and ‘presumed pathogenic’ were counted as mutations.

### Whole Transcriptome Sequencing (WTS)

FFPE specimens underwent pathology review to measure percent tumor content and tumor size; a minimum of 10% of tumor content in the area for microdissection was required to enable enrichment and extraction of tumor-specific RNA. The Qiagen RNA FFPE tissue extraction kit was used for RNA extraction, and the RNA quality and quantity were determined using the Agilent TapeStation. Biotinylated RNA baits were hybridized to the synthesized and purified cDNA targets, and the bait-target complexes were amplified in a post-capture PCR reaction. The Illumina NovaSeq 6500 was used to sequence the whole transcriptome from tumor samples to an average of 60 M reads. Raw data were demultiplexed by Illumina Dragen BioIT accelerator, trimmed, counted, excluded from PCR-duplicates, and aligned to human reference genome hg19 by STAR aligner. Transcripts Per Million Molecules (TPM) were generated using the Salmon expression pipeline, for transcript counting.

Gene set enrichment analysis was used to calculate enrichment scores of 50 Hallmark pathways between the cohorts of interest (Subramanian A, PNAS 2005; Barbie DA, Nature 2009).

### Clinical outcomes

Real-world overall survival (OS) information was obtained from insurance claims data and calculated from the time of tissue biopsy to last contact. Hazard ratio (HR) was calculated using the Cox proportional hazards model, and *P-*values were calculated using the log-rank test.

### Ethics statement

This study was conducted in accordance with the guidelines of the Declaration of Helsinki, Belmont Report, and U.S. Common Rule. In keeping with 45 CFR 46.101(b)^4^, this study was performed using retrospective and deidentified clinical data. This study was thus considered institutional review board exempt, and no patient consent was necessary.

### Cell culture

Human lung adenocarcinoma cell lines, HCC4006 (purchased from the American Type Culture Collection (ATCC, Manassas, VA)) and PC-9 (Sigma Aldrich), were cultured in RPMI 1640 media (Corning, Cat# 10040CV) supplemented with 10% heat-inactivated fetal bovine serum (FBS) (Gibco) and penicillin/streptomycin (Corning) at 37 °C, 5% CO_2_. Cells were routinely tested for mycoplasma every 6 months using the LookOut mycoplasma detection kit (Sigma Aldrich). Pools of ERO1A knockout cells were generated by Synthego with the guide sequence AUGGUUCUUACAGAUUGACA for HCC4006 cells and the guide sequence AGAAAGGACACGGCCUCUUC for PC-9 cell line by electroporating cells with Cas9 and sgRNAs. Single-cell derived clones were produced by flow cytometry sorting of single cells per well of a 96-well plate and expansion into clonal populations. Control cells were electroporated with non-targeting sgRNAs and Cas9.

LOXL2 and LAMC2 knockdown cells were generated using lentiviral pSMART hCMV-TurboGFP shRNA expressing plasmids (Horizon Discovery, Cat# V3SH11240-230463535 and V3SH11240-230789509, respectively). Empty vector was used as a control. Lentiviral particles were generated as follows: HEK293T cells (ATCC) were transfected with pSMART vectors and lentiviral packaging plasmids pCMV-VSV-G and psPAX2 (Addgene) using Turbofect (Thermo Scientific). The resulting lentivirus supernatant was collected, centrifuged, and filtered through 0.45 µm filters (Millipore). Stable cell lines expressing shRNA or control vectors were generated by lentiviral infection in the presence of hexadimethrine bromide (Sigma Aldrich) and were selected by treating cells with puromycin (InvivoGen).

For generation of conditioned media, 500,000 cells were plated in 10 ml of RPMI 1640 media onto 10 cm dishes. Fourty-eight hours later, conditioned medium was collected into 15 ml centrifuge tubes, centrifuged at 1200 rpm for 10 min, supernatants were transferred to new tubes and stored at −80 °C for later use.

### Immunoblotting

Cells were lysed in RIPA buffer (Millipore) with addition of protease and phosphatase inhibitors (Millipore) or with Laemmli buffer (62.5 mM Tris-HCl pH 6.8, 2% SDS, 10% glycerol). After measuring total protein concentration using BCA assay (Pierce), 30–50 µg of protein were separated by SDS-PAGE and transferred to a polyvinylidene fluoride (PVDF) membrane (Millipore). Blots were blocked with 5% non-fat milk in TBST (tris-buffered saline with 0.1% Tween-20) and incubated with primary antibodies diluted in antibody diluent solution (Invitrogen, Cat# 003218). After incubation with HRP-conjugated secondary antibodies (Jackson ImmunoResearch), immunocomplexes were detected using enhanced chemiluminescence (Millipore) in an Amersham 680 imager (Cytiva). Densitometry was performed using an Amersham 680 imager software and protein levels were normalized to β-actin or GAPDH levels. Conditioned medium with addition of protease and phosphatase inhibitors was concentrated by centrifugation in Amicon Ultra filters with 10 kDa molecular weight cut off (Millipore) and then processed as described above. Antibodies used are provided in Table 1.

**Table 1:** Antibodies used for Western blot experiments

Antigen	Company	Catalog number
ERO1A	Santa Cruz	sc-365526
ERO1B	Thermo Scientific	PA5-25142
GAPDH	Sigma Aldrich	G8795
EGFR	Cell Signaling Technology	4267 P
p-EGFR	Cell Signaling Technology	3777S
Beta-actin	Sigma Aldrich	A5316
LAMC2	Thermo Scientific	MA5-24646
LOXL2	Thermo Scientific	PA585210
PLOD2	Cell Signaling Technology	44709
ATF4	Cell Signaling Technology	11815S
BiP	BD Biosciences	610978
PDI	Novus Biologicals	NB300-517
p-eIF2	Cell Signaling Technology	3398 T
eIF2	Cell Signaling Technology	5324S

### Soft agar and colony formation assays

500 cells/well were plated in 500 µl of 0.5% low melting temperature agarose (Lonza) onto 12-well plates coated with 1 ml of 1% low melting temperature agarose and were allowed to grow for 14 days. Colonies were stained with aqueous 0.05% Crystal Violet, containing 20% methanol, imaged on the Olympus MVX microscope, and quantified using ImageJ. For clonogenic growth assay on plastic plates, 500 cells/well were plated onto 12-well plates. After 10 days, cells were fixed in 100% methanol, stained with 0.5% Crystal Violet, imaged on the Olympus MVX microscope, and colonies were enumerated using ImageJ. All experiments were performed in triplicates and repeated at least 3 times.

### Tumor sphere formation assay

3000 cells per well were embedded in 0.9% (HCC4006 cells) or 6000 cells/well were embedded in 1.9% (PC-9 cell line) methyl cellulose (MethoCult, Cat# H4100, Stem Cell Technology), supplemented with 20 ng/mL bFGF (Gibco), B27 Supplement (Gibco), and 20 ng/mL EGF (Sino Biological), and plated in triplicates in 24-well ultralow attachment plates (Nunclon Sphera, Thermo Scientific). Tumor spheres were allowed to grow for 10–14 days, imaged on the EVOS FL Auto 2 imager (Life technologies), and quantitated using ImageJ software.

### Analysis of publicly available proteomics data

The tumor proteome dataset from lung adenocarcinomas^27^ analyzed by the NCI CPTAC and processed through their pipeline was analyzed using LinkedOmics (http://linkedomics.org/data_download/CPTAC-LUAD/)^28^to calculate Pearson correlations between ERO1A and ERO1B and the rest of the proteins in the dataset. Data were exported and sorted in Microsoft Excel.

### Immunofluorescent staining of tumor spheroids

5000 HCC4006 control or ERO1A knockout cells per well were plated into ultra-low attachment U-shaped-bottom 96-well plates (Nunclon Sphera, Thermo Scientific). Spheroids were allowed to form for 2 days and were loaded into 3D Flowchips (Protein Fluidics) and stained in the Pu MA System (Protein Fluidics) according to the manufacturer’s instructions. Briefly, spheroids were fixed in 4% paraformaldehyde, washed in phosphate buffered saline (PBS), blocked with 1% BSA in PBST and incubated with primary antibodies diluted in blocking solution overnight at room temperature. The primary antibodies were LOXL2 (Thermo Scientific, Cat# MA5-24646) and LAMC2 (Thermo Scientific, Cat# PA-585210). After incubation with fluorescently stained secondary antibodies (Thermo Scientific), spheroids were counterstained with Hoechst 33342 (Thermo Scientific) and imaged on the confocal Nikon A1R microscope. Integrated density of fluorescence staining was measured in ImageJ.

### Animal study

All animal experiments were performed in accordance with Guidelines for Animal Experiments at West Virginia University with the approval of the Institutional Animal Care and Use Committee (IACUC). 2 million of either PC-9 control or PC-9 ERO1A knockout cell line were injected via tail vein into SCID-Beige mice Charles River Laboratories. Mice were euthanized according to the ACUC guidelines (Carbon dioxide (CO2) asphyxiation followed by cerical dislocation) and decision for euthanasia was based on clinical end points established by the IACUC protocol. Upon euthanasia, mouse lungs were removed, fixed in formalin, and paraffin embedded. H&E stained slides with full lung sections were scanned using the Olympus VS120 Slide Scanner. Tumor area was quantitated in 5 randomly selected mice/group by a blinded researcher using ImageJ.

### ELISA

LAMC2 levels in conditioned medium were detected using human LAMC2 ELISA kit (Abcam, Cat# ab282301) according to the manufacturer’s instructions. Conditioned medium from 3 independent experiments in triplicates was used.

### Statistical analysis

was performed in GraphPad Prism. Results are presented as means ± standard deviation. Differences were considered statistically significant when the *p*-value < 0.05.

### MTT assay

Cells (3000 PC-9 and 5000 HCC4006 per well) were allowed to adhere overnight in 96 well plate prior to exposure to varying concentration of Osimertinib. The vehicle control consisted of final concentration of 0.02%. Following 72 h of drug exposure.

50 µl of MTT (2 mg/ml) solution was added in all the wells and incubated for 1 h at 37°C. Subsequently, all the solution along with the media in 96 well plates were aspirated and 200 µL of DMSO. The percent survival was calculated in relation to the 0.02% treated DMSO cells for each cell line.

## Discussion

Experimental data continues to support ERO1A as an attractive target for the treatment for cancer. This evidence includes several studies indicating that *ERO1A* is a poor prognostic indicator of survival across multiple tumor types^1,3,29,30^. In addition, studies using genetic strategies to reduce or ablate the expression of ERO1A in cancer cell line models have demonstrated that a reduction in ERO1A levels inhibits growth and metastasis in vivo^15,16^. The mechanism underpinning the dependency of cancer cells on ERO1A expression appears to be multifactorial, with the cause and effect of downstream targets mediating the observed phenotype not fully elucidated. Reducing ERO1A expression appears to slow down oxidative protein folding, but due to redundancy of other oxidoreductases present in the ER, including ERO1B, reducing the expression of ERO1A is not sufficient to ablate oxidative folding in the ER. Interestingly, depletion of ERO1A was shown to result in increased N-glycosylation and inhibition of secretion of VEGF^17^ suggesting that a subset of proteins may be dependent on ERO1A oxidative folding for efficient secretion. Despite the redundancy in oxidative protein folding within the ER of mammalian cells, cancer cells appear to have an increased dependency on ERO1A which maybe more appreciable in the context of the tumor microenvironment or potentially drug response. In support of this point, *EGFR*^MUT^-LUAD cells continue growing despite the absence of ERO1A expression in cell culture conditions. However, phenotypes associated with depletion of ERO1A were more notable in the context of spheroid or clonogenic growth in soft agar, drug response or growth in vivo.

In this manuscript, we focused on the role of potential downstream targets that associated with ERO1A protein expression using the CPTAC data set generated from LUAD patients to guide the selection of targets. To our surprise, multiple extracellular matrix proteins showed increased expression in the context of higher ERO1A expression. This pathway was of interest to our laboratory, as we and others have shown that cell adhesion to matrix is sufficient to induce a multi-drug resistant phenotype via a multitude of mechanisms, including decreased expression of Bim, augmentation of IL-6 signaling and activation of the JAK-STAT3 pathway and increased expression of p27^31–36^. Interestingly, using patient-derived proteomic data, we found a reduction in LAMC2 and LOXL2 secretion when ERO1A was expressed at lower levels and confirmed these observations in EGFR-driven cells depleted of ERO1A. Additional studies are warranted to determine whether this is due to increased N-glycosylation, which was recently reported to occur with VEGF in cells depleted of ERO1A^17^. The decrease in LAMC2 and LOXL2 secretion correlated with other phenotypic changes, including decreased clonogenicity, tumor sphere formation, spheroid growth, growth in vivo and increased drug sensitivity. However, we were not able to rescue all phenotypes with conditioned media derived from the parental cell line. Together, these data indicate that ERO1A may contribute to a combination of secreted and non-secreted factors or that deposited matrix, which is not captured in the conditioned media, contributes to the observed phenotypes.

Our data indicate that ERO1A is an attractive target for the treatment of *EGFR*^MUT^-LUAD in combination with EGFR inhibitors. However, it is currently unclear whether expression of ERO1A will be sufficient to determine whether a tumor is sensitive to treatment with an ERO1A inhibitor. Considering data showing that ERO1A is not a statistically significant independent marker of prediction of response to Osimertinib treatment it is likely that additional understanding of downstream dependency or phramcodynamic markers will be required to predict sensitivity to inhibition of ERO1A activity as a single agent or in combination with Osimertinib with respect to tumor response. *ERO1A*-deficient mice are viable, indicating that ERO1A targeting has potential efficacy for the treatment of cancer, which is further strengthened by the relatively mild phenotype associated with *ERO1A* KO mice^25^. The cardiomyocytes from *ERO1A* KO mice demonstrated reduced peak calcium flux induced by adrenergic stimulation and were sensitized to adrenergic blockade^37^, and *ERO1A* KO mice were protected from progressive heart failure using a transaortic constriction model^37^. Moreover, double-knockout of both *ERO1A* and *ERO1B* in mice is not lethal, suggesting that targeting both enzymes is feasible from a toxicity perspective^25^. Similar to our findings in NSCLC cell lines, secretion of ECM proteins is reduced in the homozygous *ERO1A* KO, and this reduction can be detected using Masson Trichrome staining in vivo^25^. The *ERO1* KO mouse phenotype with respect to matrix staining is further increased when deleting the paralog *ERO1B*, as well as *PRDX4*. Together, data generated from mouse KO models suggest that a therapeutic window will be achievable with specific pharmacological inhibition of ERO1A for the treatment of cancer, and that inhibition may reshape the TME landscape. Similar to kinase inhibitors, a challenge to the development of ERO1A inhibitors is off-target activity against other FAD-containing enzymes. We recently reported that T151742 demonstrates increased specificity and potency over the commercially available compound called EN460^2,38^. However, more specific and potent inhibitors are warranted to fully pharmacologically validate ERO1A as a target for the treatment of cancer, which is a current focus of our laboratory.

## Supplementary information

Supplemental Figures

## Data Availability

The datasets generated during and/or analyzed during the current study are available from the corresponding author on reasonable request. The de-identified sequencing data cannot be publicly shared due to the data usage agreement between the facilities of the study team. Qualified researchers can apply for access to these summarized data by contacting N.G. and signing a data usage agreement. The processed NGS data are available at: (summary table of primary tumors to match figures attached). Other questions regarding the data of this study are welcomed on request to the corresponding author, L.A.H.
