# Supplementary material for: ERO1A levels are a prognostic indicator in EGFR mutated non small cell lung cancer
Source: NPJ Precis Oncol. 2024 Nov 4;8:250. doi: 10.1038/s41698-024-00736-1 (PMC11535241; doi:10.1038/s41698-024-00736-1)

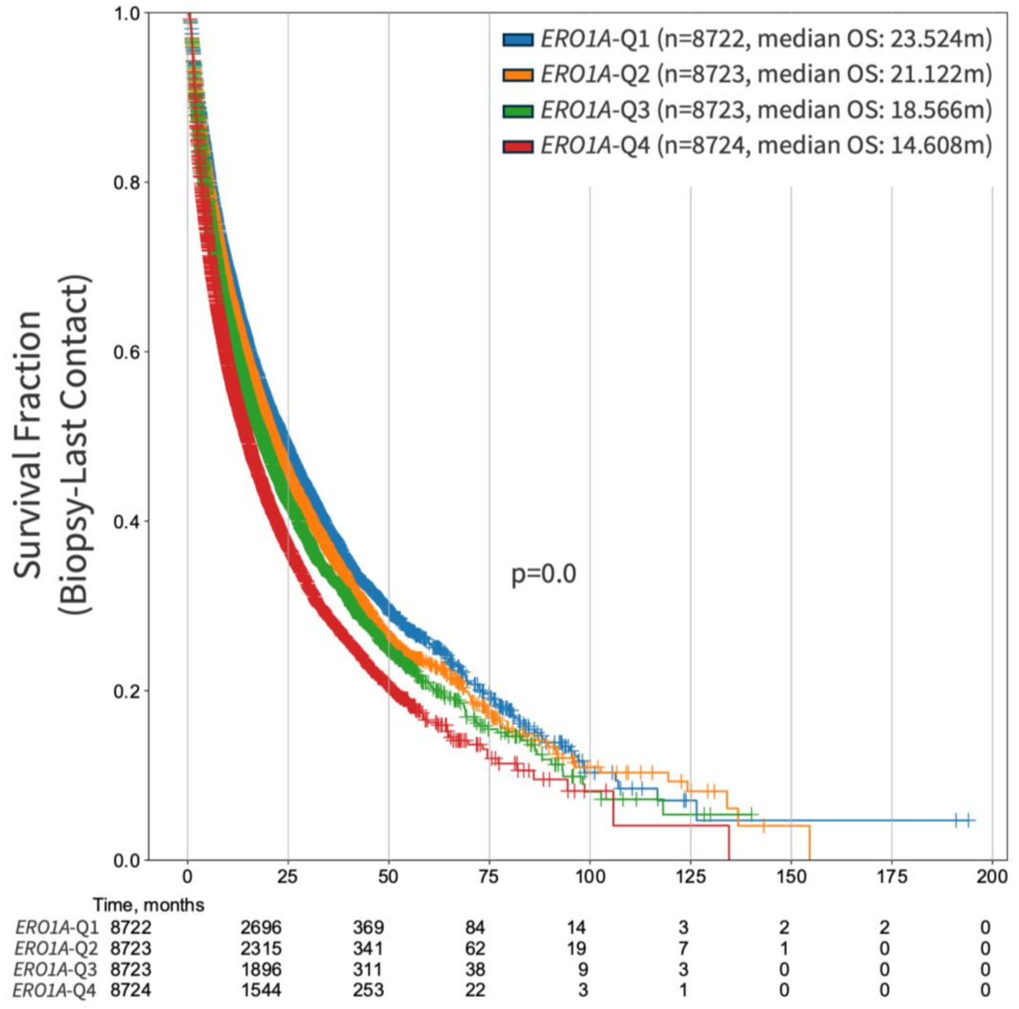

Supplemental Figure 1: NSCLC specimens were stratified into quartiles based on ERO1A expression (Q1:Lowest, Q4:Highest) and its association with overall survival (OS) was assessed.

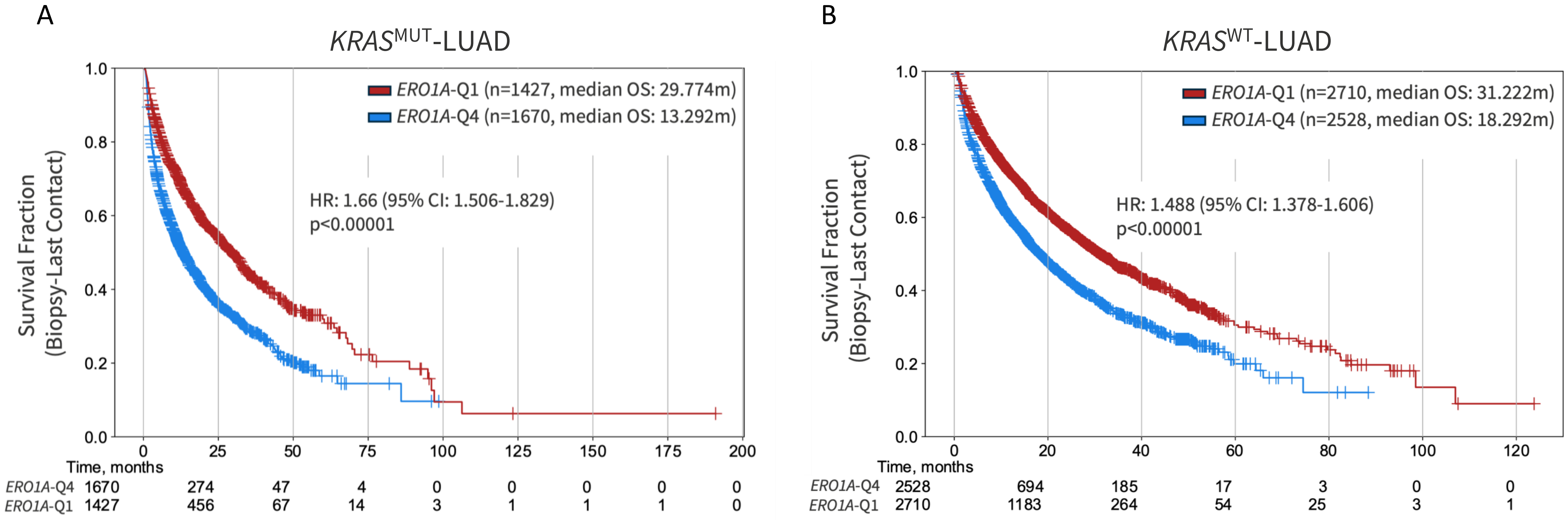

Supplemental Figure 2: The association of *ERO1A* expression with OS in LUAD were assessed in the context of *KRAS* mutation status (A) *KRAS*^{MUT} & (B) *KRAS*^{WT}. For the analysis, tumors were stratified based on high and low quartile expression of *ERO1A,* i.e. *ERO1A*-Q4 and *ERO1A*-Q1 respectively.

ERO1A Expression Protein Level

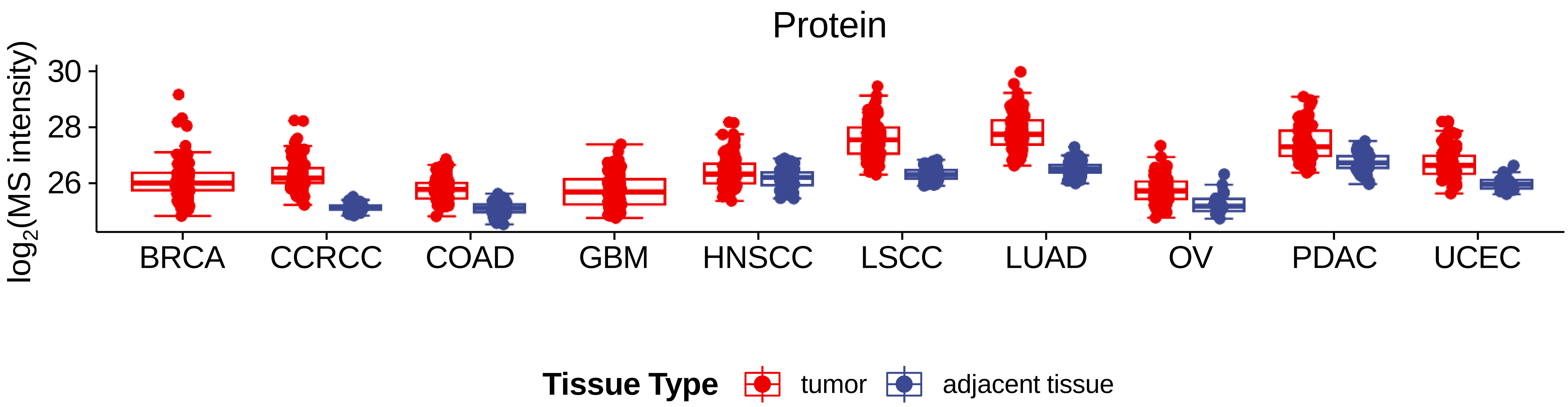

Supplemental Figure 3: Data from previous Clinical Proteomics Tumor Assessment Consortium (CPTAC) projects were mined to examine levels of ERO1A protein expression across 10 tumor types (**B**). Levels in tumor (**red**) are compared to adjacent tissues (**blue**), and lung tumors were among the tissues with highest expression. Queries were made using the LinkedOmics Knowledge Base (https://kb.linkedomics.org/).

source: linkedomics

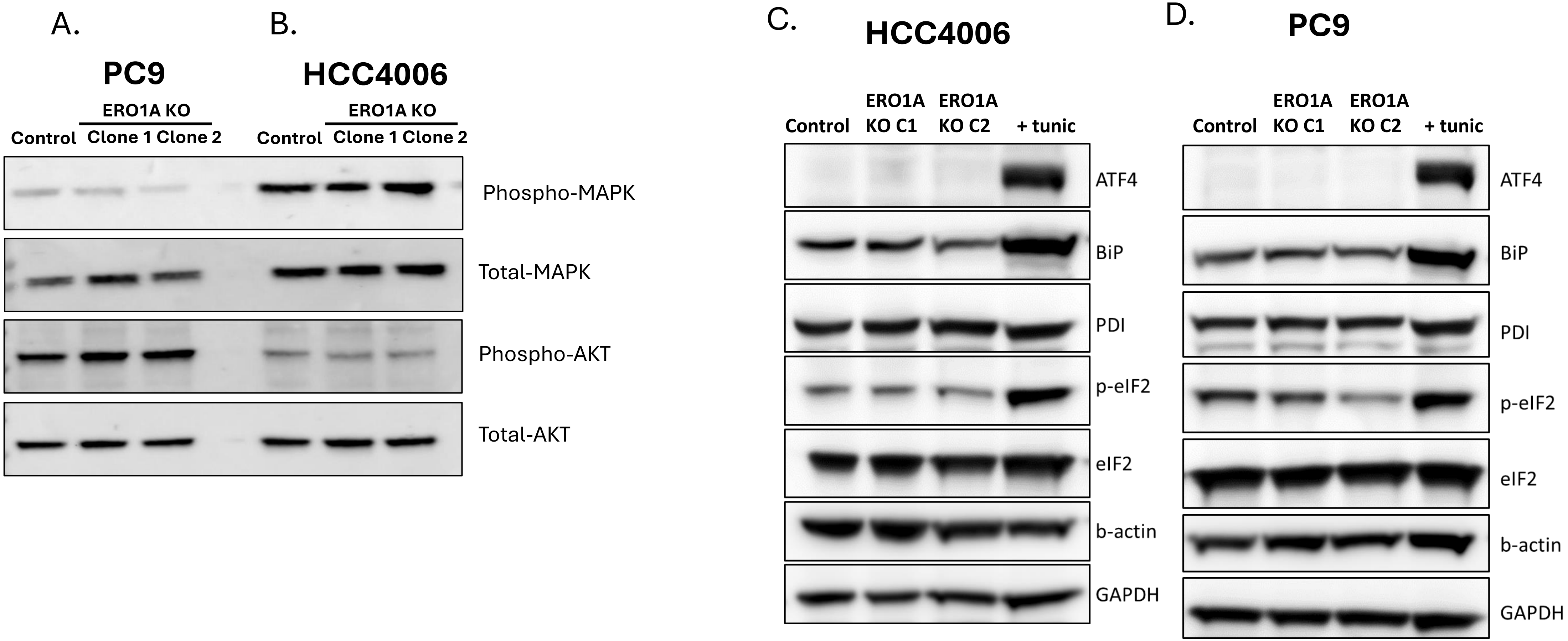

Supplementary Figure 4: A-B: ERO1A KO cells showed no changes in pAKT or pMAPK compared to control cells. C-D: Control and ERO1A KO cells showed no changes in ER stress markers. Positive control consisted of PC-9 cells treated with 2 μM tunicamycin for 18 hours

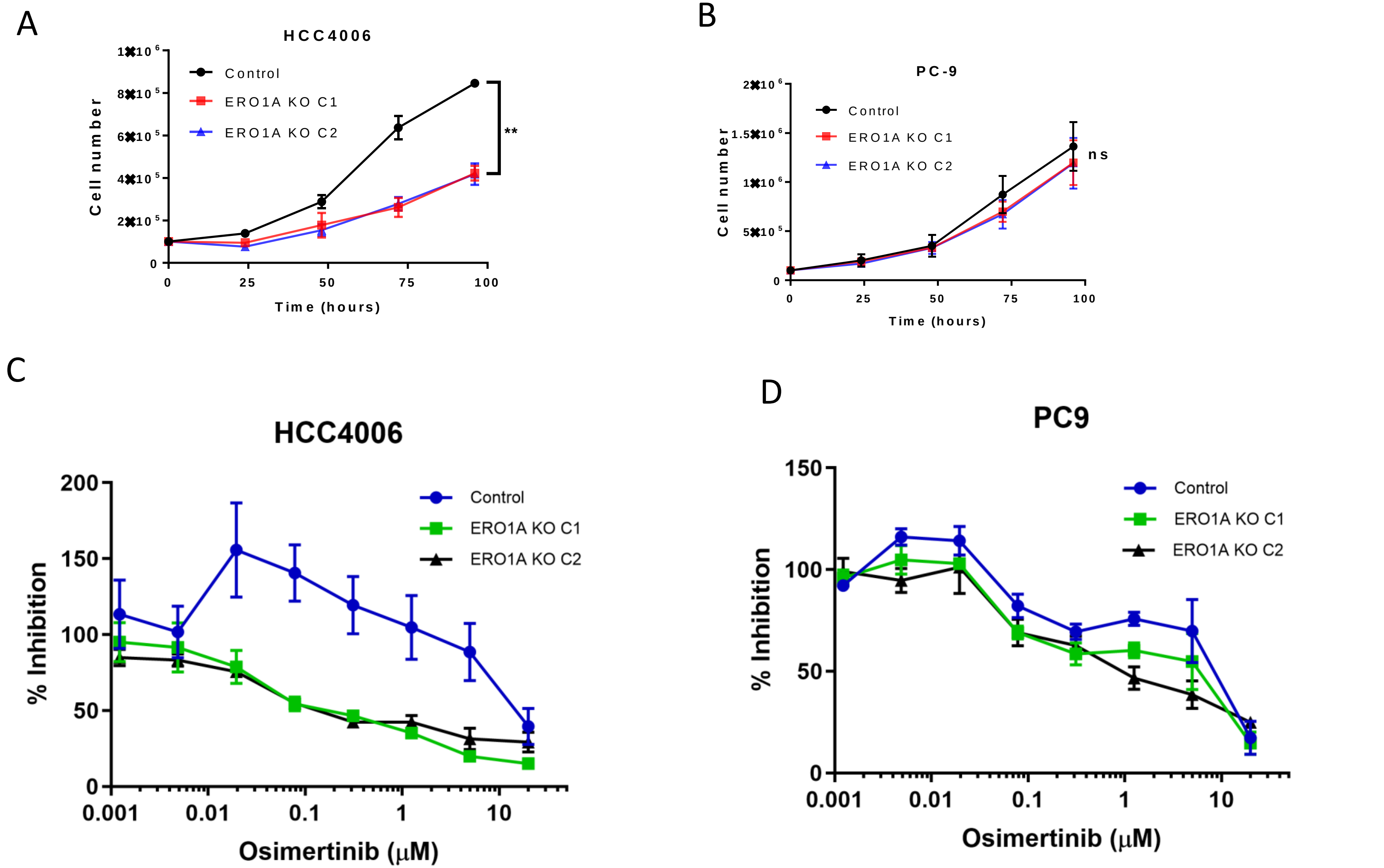

Supplemental Figure 5: A-B: Growth was determined by seeding cells at 50,000 and counting cells at the indicated time points n=3 independent experiments. HCC4006 cell depleted of ERO1A showed a significant ($p<0.05$ two-way repeated ANOVA) while PC-9 cells showed a trend but was not significant ($p>0.05$, two-way repeated ANOVA. **C-D:** Inhibition to Osimertinib treatment was determined by MTT analysis. N=4 independent experiments. The mean IC50 value for HCC4006 was 6.75 μM; HCC4006 ERO1A KO C1 0.05 μM and HCC4006 KO C2 0.07 μM . PC-9 IC50 value was 5.0 μM ; PC-9 ERO1A KO C1 1.9 μM and PC-9 ERO1KO C2 0.22 μM.

A

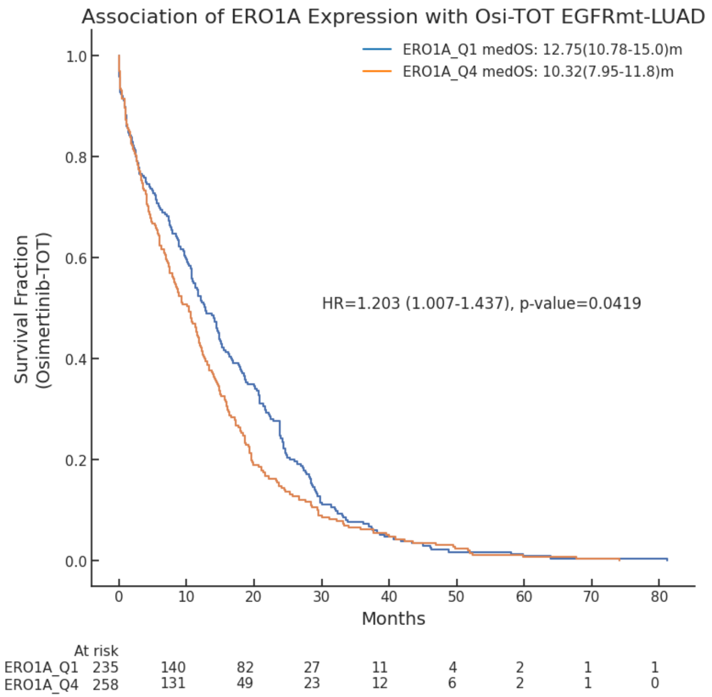

B

Molecular Alterations	% Prevalence ERO1A Q4	% Prevalence ERO1A Q1	p-value	q-value
Mutations				
TP53	73.4	49.62	0	0
CTNNB1	3.38	8.75	0.0072	0.0101
APC	1.35	4.56	0.023	0.0308
Amplifications				
EGFR	14.92	6.2	0.001	0.0015
CDK6	2.74	0	0.0078	0.0108
MYC	4.07	0.78	0.0139	0.019
NIN	2.1	0	0.0232	0.031
IO Related Biomarkers				
PD-L1+ (22c3)	60.14	34.12	0	0

C

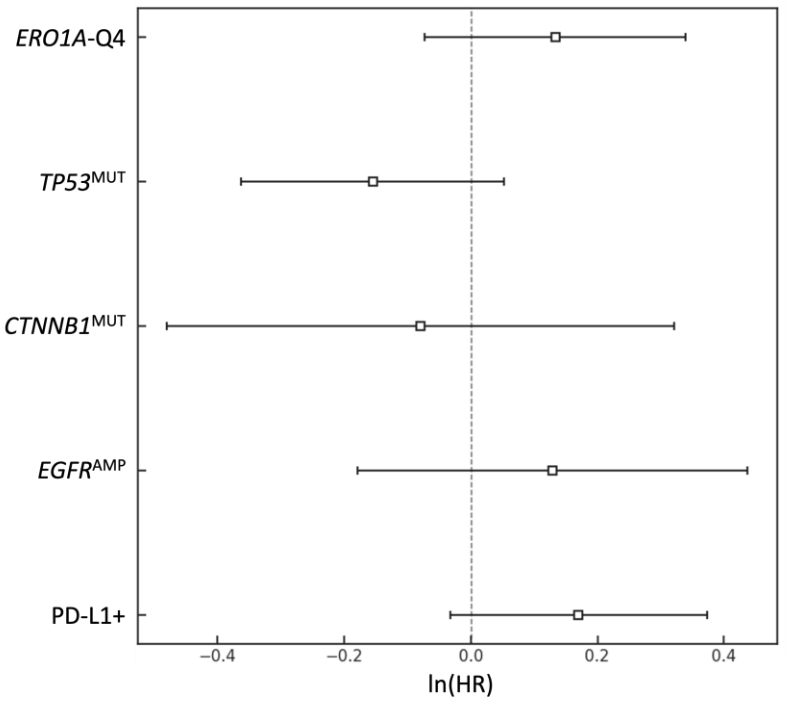

Supplemental Figure 6: Multivariate analysis to test the association of ERO1A expression with Osimertinib-TOT. ERO1A quartile cutoffs were determined based on the expression of ERO1A in EGFRmt-LUAD. (A) Patients with tumors expressing high ERO1A experienced a shorter time on Osimertinib treatment. (B) Molecular alterations associated with ERO1A expression prevalent in either cohort at >=2% and statistically significantly different are shown in the table. (C) Molecular alterations with a prevalence difference of at least 5% between the cohorts were incorporated as co-variates in a complete-case multivariate analysis. None of the co-variates including ERO1A (p=0.2) were independently associated with Osimertinib-TOT. n=447

Supplementary figures- uncropped and unprocessed scans of blots

Figure 4.A (PC-9)

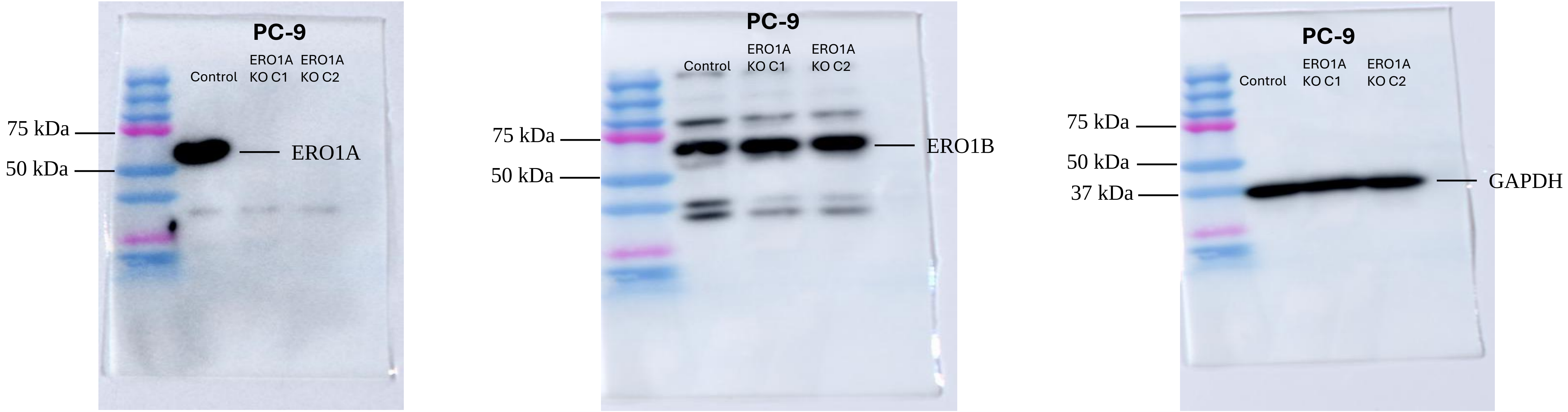

Figure 4.A (HCC4006)

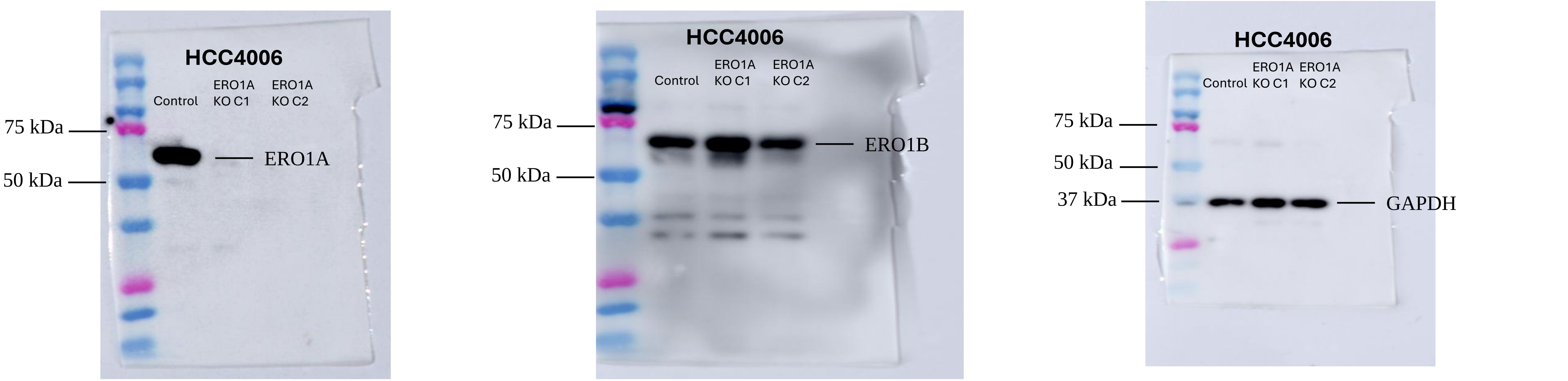

Figure 4.A (PC-9 Cell line)

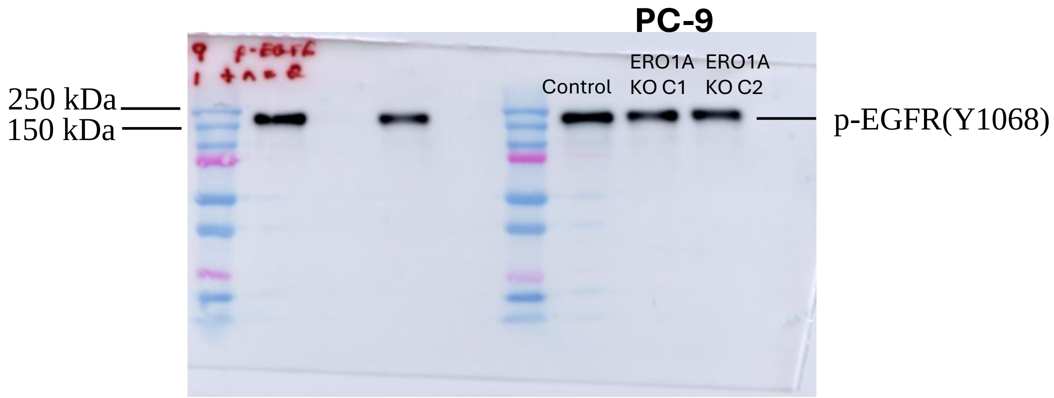

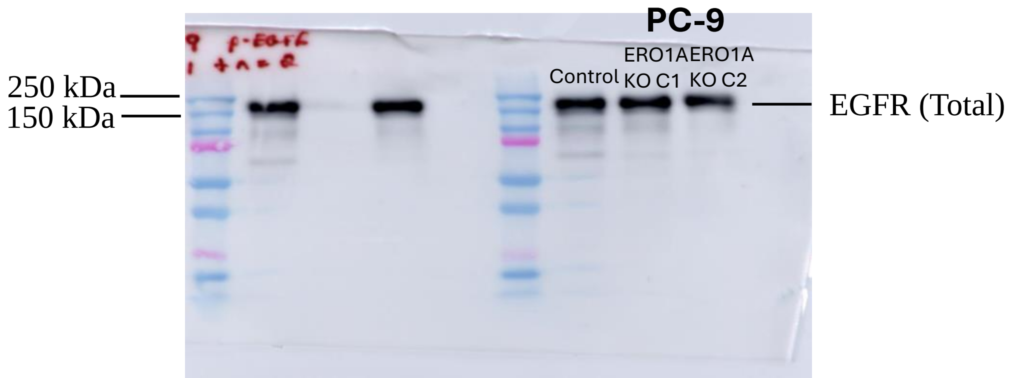

Total-EGFR Figure 4.A (PC9 Cell line)

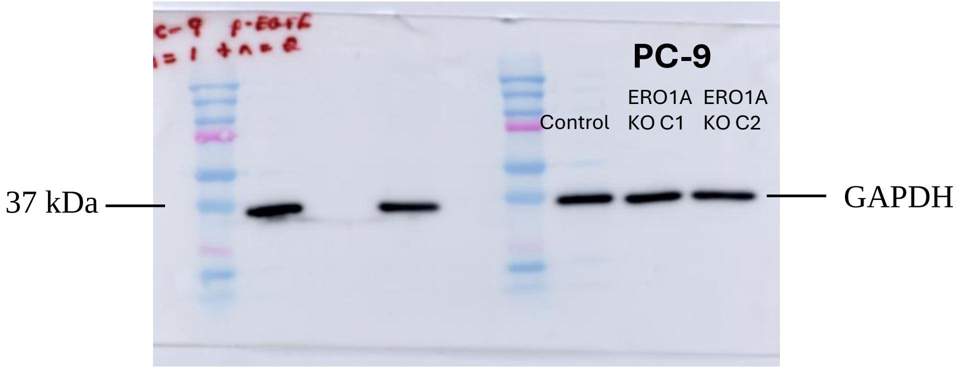

Figure 4.A (HCC4006 Cell line)

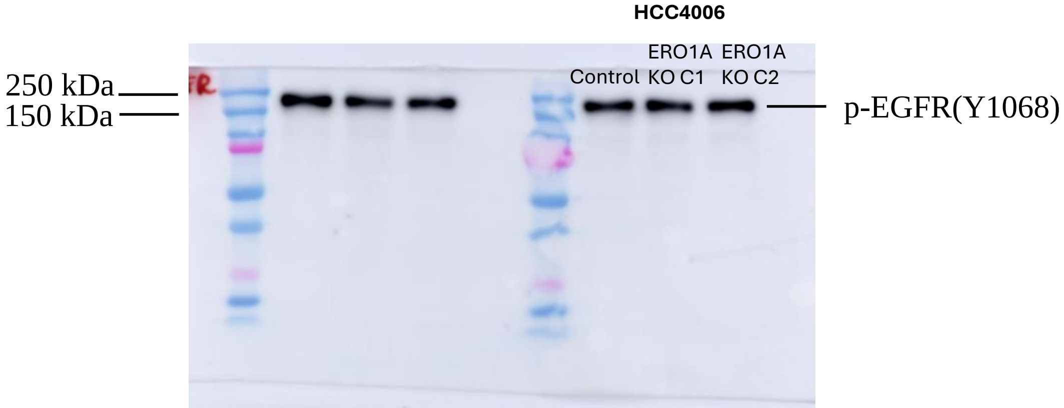

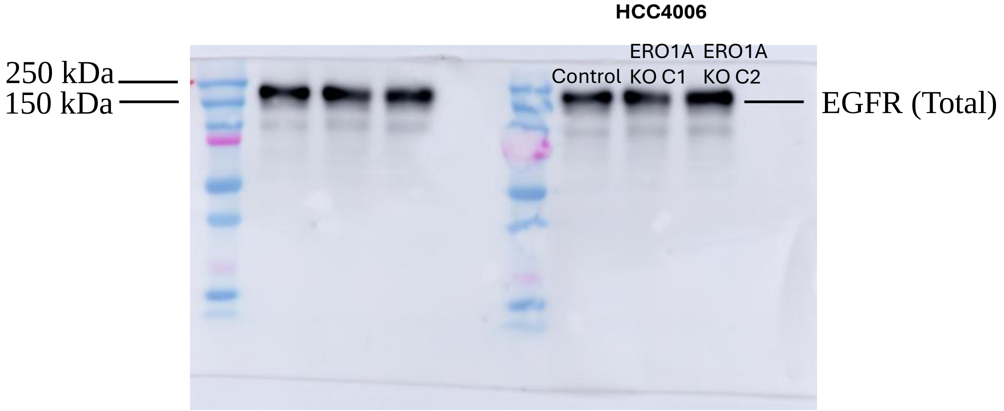

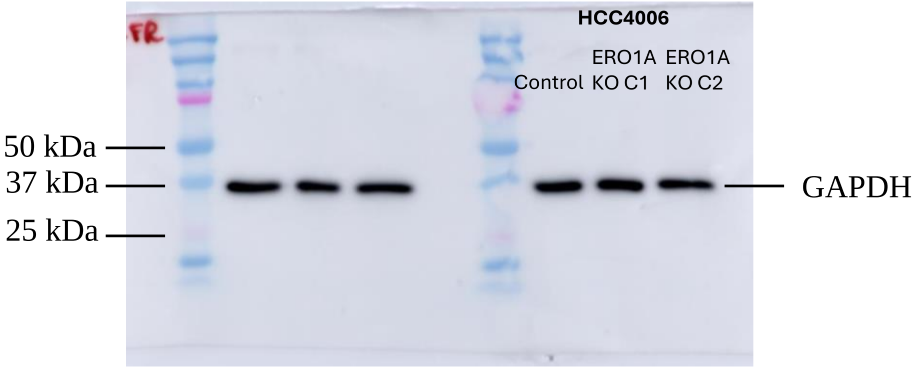

Figure 5.A (Whole cell lysate)

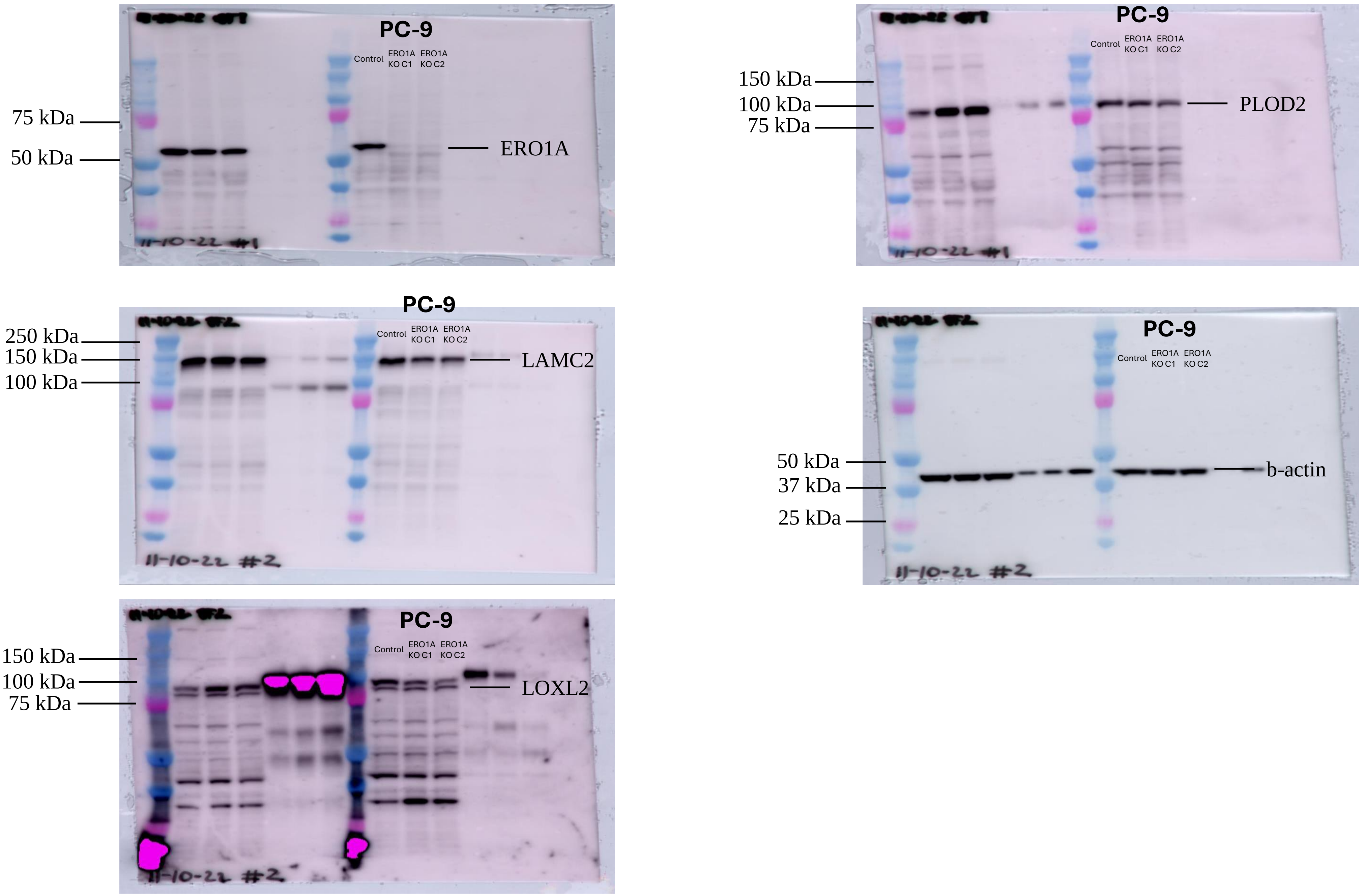

Figure 5.A (Conditioned medium)

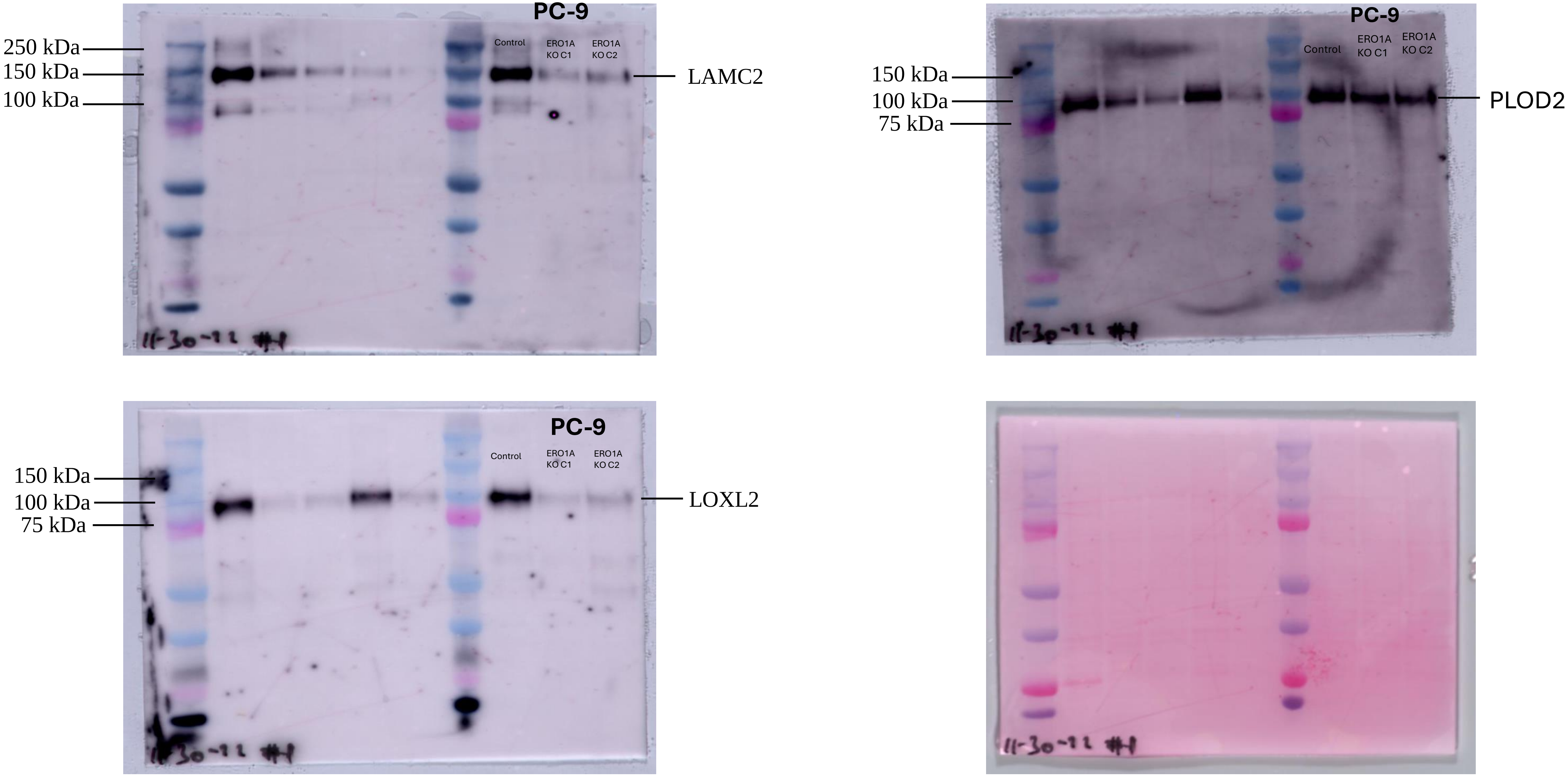

Ponceau S

Figure 6.C (Whole cell lysate)

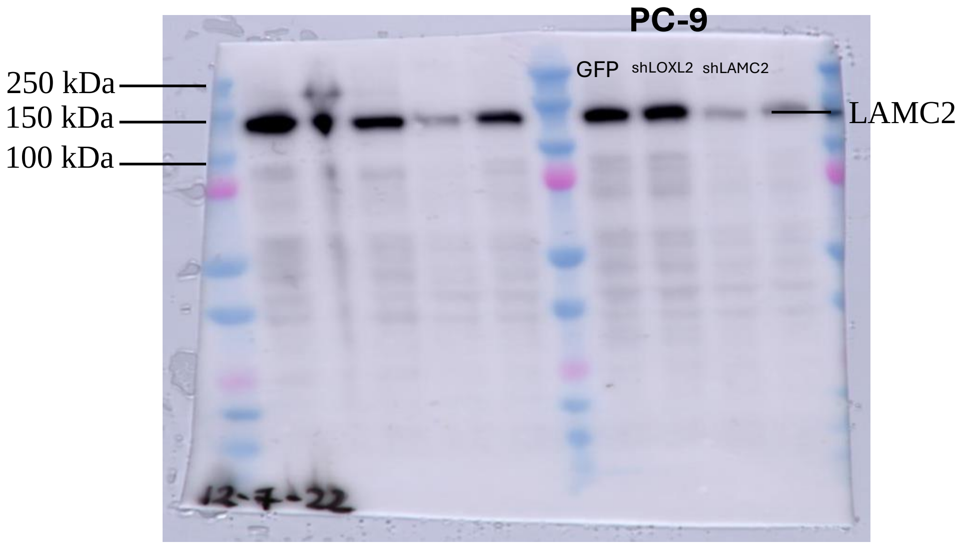

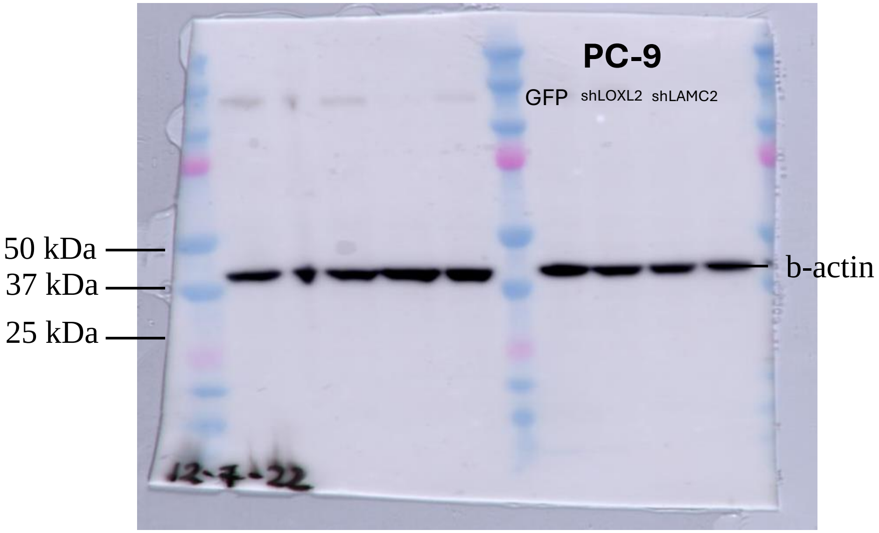

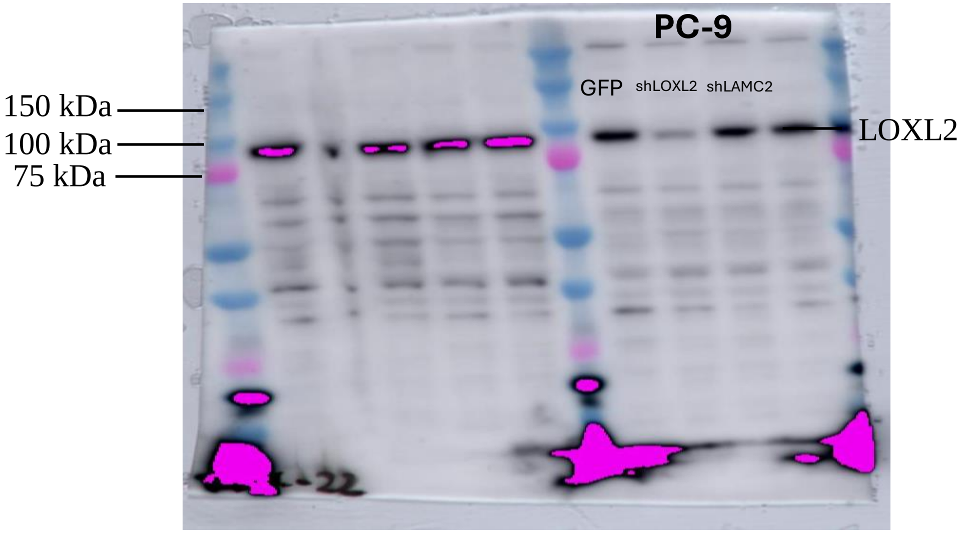

Figure 6.C (Conditioned medium)

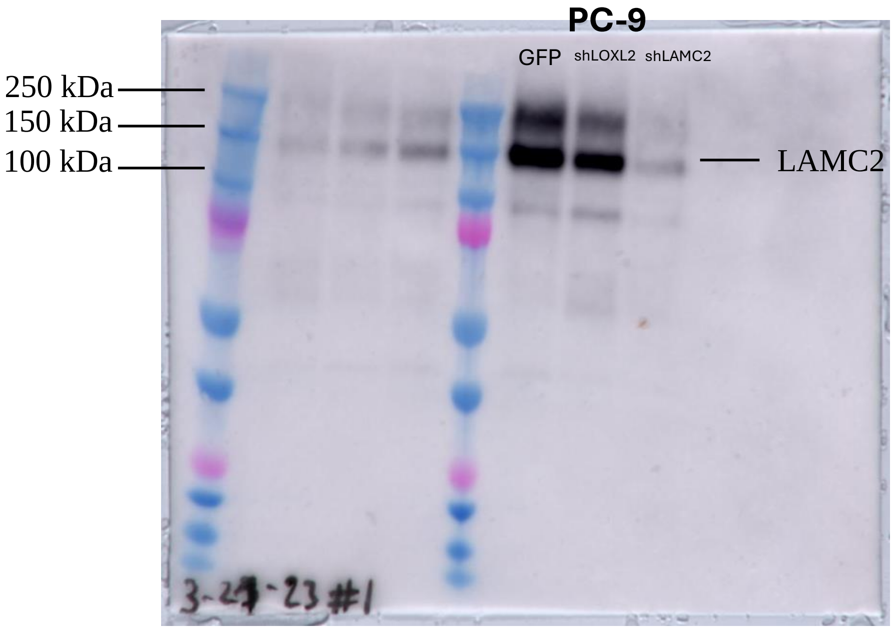

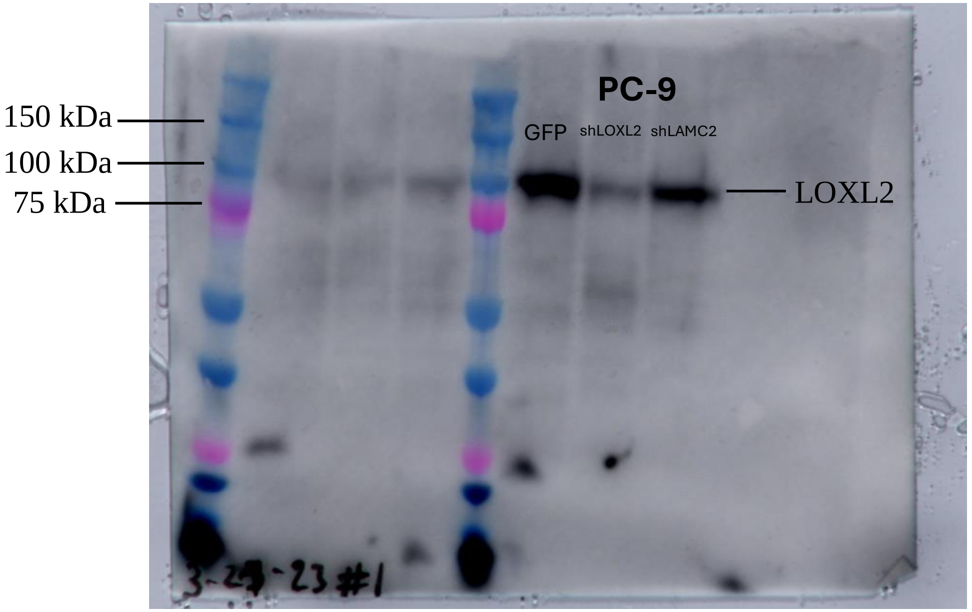

Supplement: Supplementary file 1 — Supplemental Figures [file 41698_2024_736_MOESM1_ESM.pdf]
